# Missing harmonic dynamics in generalized Snell’s law: revealing full-channel characteristics of gradient metasurfaces

**DOI:** 10.1038/s41377-025-02009-3

**Published:** 2025-09-15

**Authors:** Yueyi Zhang, Fengyuan Han, Yibing Xiao, Ziwen Zhang, Jitao Yang, Yulu Lei, Fei Gao, Hongsheng Chen, Chao-Hai Du

**Affiliations:** 1 Center for Carbon-Based Electronics, School of Electronics, Peking University, Beijing, 100871 China; 2 State Key Laboratory of Photonics and Communications, School of Electronics, Peking University, Beijing, 100871 China; 3 Key Lab. of Advanced Micro/Nano Electronic Devices & Smart Systems of Zhejiang, College of Information Science and Electronic Engineering, Zhejiang University, Hangzhou, 310058 China

**Keywords:** Sub-wavelength optics, Nanophotonics and plasmonics, Metamaterials

## Abstract

The conventional generalized Snell’s law (GSL), derived from classical laws of optical reflection and refraction, governs wavefront manipulation via phase gradients but neglects higher-order spatial harmonics inherently excited by the mutual coupling among meta-atoms on a metasurface. Here, we introduce a spatial harmonic-expanded GSL (SH-GSL) framework by unifying phase-gradient control with Floquet periodicity, establishing spatial harmonics as independent degrees of freedom rather than conventional parasitic disturbances. The SH-GSL framework rigorously identifies the intrinsic harmonic dynamics inherent to metasurfaces, which is a critical feature absent in GSL. Furthermore, this framework further reveals that all gradient-phase metasurfaces inherently function as multichannel platforms due to full spatial harmonics, with this multifunctionality rooted in nonlocal Floquet-Bloch modal interactions. Experimental validation demonstrates: abnormal spatial-harmonic reflection with angular precision ( < 5° deviation), multi-beam splitting (dual/quad configurations) via the relationship between specific harmonics and compensation wave vectors, and a perfect three-channel retroreflector achieving up to 99% efficiency, where parasitic harmonics are confined to near-field plasmonic regimes. This framework establishes a deterministic Floquet-engineered momentum compensation mechanism to simultaneously activate target harmonic channels while confining parasitic harmonics to near-field plasmonic regimes. Experimental validation confirms the framework’s accuracy and scalability, bridging momentum-space physics with practical meta-plasmon systems. This work redefines metasurface engineering paradigms, unlocking advancements in ultra-dense beamforming, sensing, and meta-photonics through harmonic-division multiplexing.

## Abstract (web summary)

SH-GSL combines gradient metasurfaces and Floquet theory to control fundamental and high-order spatial harmonics, enabling abnormal reflection, multi-beam splitting, and perfect multi-channel retroreflection.

## Graphical abstract

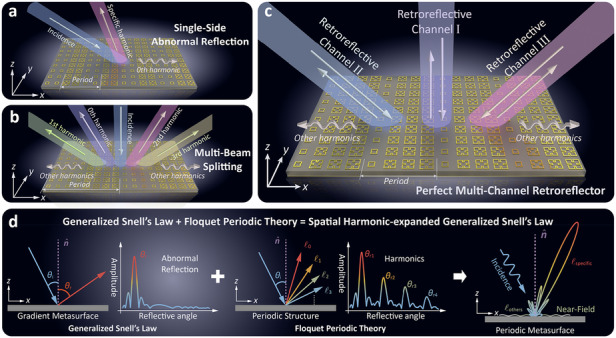

## Introduction

The advent of metasurfaces has revolutionized wavefront engineering by enabling precise control of electromagnetic waves through subwavelength structures^1–8^. With the advantage of the ultra-thin thickness and excellent manipulation ability, many fascinating and unique functionalities have been realized by utilizing metasurfaces, such as anomalous refraction and reflection^9–12^, meta-lens focusing^13–18^, polarization transformation^19–22^, perfect absorption^23–27^, etc. With the development of the next generation of mobile communication technology, higher demands are put forward for the miniaturization and integration of devices than before. Thus, future electromagnetic devices must meet the requirements of miniaturization, integration, multi-functionality, generalization, and scalability. With the advantages of integral and miniaturizable characteristics of metasurfaces, the investigation of meta-devices is a significant trend^28,29^.

The generalized Snell’s law (GSL), proposed by Capasso and colleagues in 2011, laid the foundation for predicting anomalous reflection and refraction angles by linking abrupt phase gradients to momentum conservation^30^. However, GSL inherently focuses on the fundamental spatial harmonic, treating higher-order harmonics as parasitic effects. This limitation has constrained metasurfaces to single-channel functionalities, such as conventional beam steering or focusing^31–41^, while multi-channel applications—ranging from holographic multiplexing to ultra-dense beamforming—demand systematic exploitation of higher-order spatial harmonics.

Recent attempts to harness higher-order harmonics have relied on numerical optimization or multi-resonant meta-atom designs^13,15^. Coding meta-atoms, leveraging convolution processing algorithms and antenna element analysis, are employed to control multi-order spatial harmonics but are limited to normal incidence scenarios^42–44^. In contrast, metagratings, composed of periodic, sparse, and polarizable particles, offer an efficient alternative for wavefront manipulation by aligning propagating Floquet modes with desired harmonic components, differing fundamentally from the transverse momentum superposition in phase-gradient metasurfaces^45–47^. However, metagratings lack the necessary degrees of freedom to effectively excite specific high-order spatial diffraction harmonics. A critical challenge persists: the absence of a deterministic theory linking metasurface periodicity, phase gradients, and higher-harmonic properties. Without such a framework, metasurface design remains trapped in a trial-and-error paradigm, unable to fully exploit the multi-channel potential of periodic structures.

**Fig. 1:**
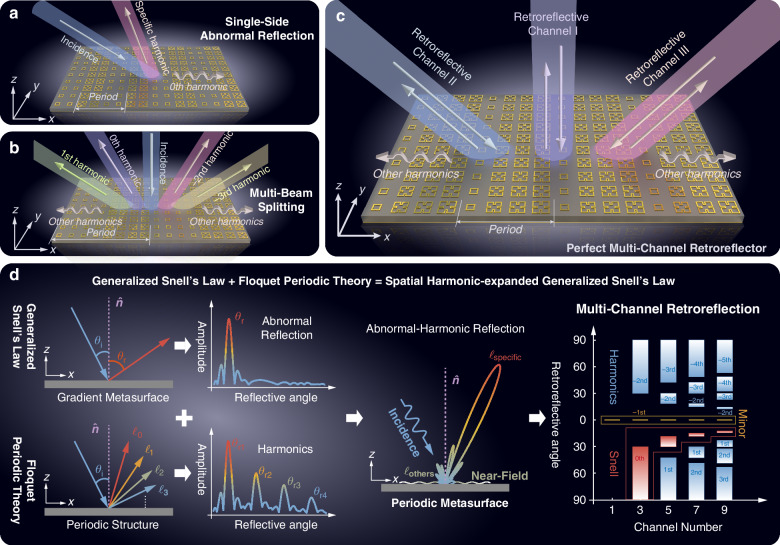
Conceptual illustration of Spatial Harmonic-expanded Generalized Snell’s Law (SH-GSL). **a** The schematic of the metasurface for abnormal-harmonic reflection. **b** The schematic of the metasurface for multi-beam splitting. **c** The schematic of the metasurface for perfect multi-channel retroreflection. **d** The illustration of the proposed SH-GSL for harmonics manipulation. The fundamental harmonic can be manipulated by gradient metasurfaces based on the generalized Snell’s law (**d** left), while high-order harmonics need to be analyzed by the Floquet period theory (**d** left). By combining the phase gradient (GSL) and the period property (Floquet period theory), the abnormal reflection of harmonics can be manipulated according to a deterministic Floquet-engineered momentum compensation mechanism of spatial harmonics (**d** middle). Furthermore, by utilizing multi-harmonics simultaneously, the multi-channel retroreflection based on multiple harmonics can be achieved (**d** right). The 0th harmonic is controlled by the generalized Snell’s law in the red box, the −1st harmonic is denoted as the mirror mode in the yellow box, and the other order harmonics can be manipulated by the Floquet period theory in the blue box

Here, we resolve this challenge by deriving a spatial harmonic-expanded generalized Snell’s law (SH-GSL) through rigorous Floquet-Bloch analysis, revealing the intrinsic harmonic dynamics in metasurfaces (Fig. 1). Our theory introduces a momentum compensation principle, where the periodicity (Floquet period analysis^48^) and phase gradient (GSL analysis^30^) of a metasurface jointly determine a compensation wave vector for each harmonic order. This principle analytically predicts anomalous angles for arbitrary harmonics via the relationship between the incident angle and the total compensation wave vector of the metasurface. Crucially, SH-GSL reveals that higher-order harmonics are not parasitic artifacts but controllable degrees of freedom governed by global periodicity. We analyze the harmonic dynamics in metasurfaces: the fundamental harmonic is manipulated by the phase gradient described in GSL, the mirror mode corresponding to the −1st harmonic cannot be controlled by metasurfaces, and the compensation wave vector can determine whether the spatial harmonics propagate in free space. Based on the designed meta-atoms, three extended applications are verified through theoretical analysis, simulations, and experiments: abnormal-harmonic reflection (Fig. 1a), multi-beam splitting (Fig. 1b), and multi-channel retroreflection (Fig. 1c). Our work redefines metasurface engineering across three dimensions:

1. From single- to multi-harmonic control: SH-GSL provides the first predictive framework for designing harmonics as independent channels.
2. From local resonance to global symmetry: Harmonic properties are dictated by periodicity and phase symmetry, decoupling design from meta-atom complexity.
3. From heuristic trial-and-error to physics-driven design: The compensation wavevector concept bridges momentum-space physics and real-world device engineering.

These advances unlock transformative applications in next-generation communications (ultra-dense beamforming). By unifying Floquet periodicity with spatial harmonics, SH-GSL signifies an era of mode-division multiplexing meta-systems, where spatial harmonics serve as parallel information carriers rather than undesired noise.

## Results

### Spatial Harmonic-expanded generalized Snell’s law

Considering the abnormal reflection scenario based on metasurfaces, the relationship between incident (*θ*_i_) and reflected (*θ*_r_) waves can be calculated according to GSL^30^:

1 $${n}_{i}{k}_{0}\,\sin \,{\theta }_{r}-{n}_{i}{k}_{0}\,\sin \,{\theta }_{i}=\frac{\Delta \varPhi }{\Delta x}$$

where *n*_i_ is the refractive index of the medium, *k*_0_ is the wave vector in vacuum, and ΔΦ/Δ*x* is the phase gradient in the transverse direction introduced by the metasurface. Substantially, the phase gradient is generated based on all the meta-atoms in the period, i.e., Δ*k*_*∥*_ = ΔΦ/Δ*x*. Then, the equation above in the case of *n*_i_ = 1 can be further written as:

2 $${k}_{//}^{r}={k}_{0}\,\sin \,{\theta }_{r}=\Delta {k}_{//}+{k}_{0}\,\sin \,{\theta }_{i}$$

where ${k}_{//}^{r}$ represents the transverse wave vector of reflected waves. From the above analysis, it can be seen that GSL expresses the intrinsic phase discontinuity of adjacent meta-atoms in each period.

It is worth mentioning that since it ignores the existence of spatial harmonics, only the abnormal reflective or refractive angle of the fundamental harmonic can be calculated by GSL. Therefore, only the simple and single-beam functional meta-device can be achieved according to GSL. From the perspective of the manipulation of multi-harmonic and the design of complex functional meta-devices, it is crucial to introduce spatial harmonics into GSL to address the manipulation of spatial harmonics. In this paper, we provide three application examples by introducing spatial harmonics, including abnormal-harmonic reflection (Fig. 1a), multi-beam splitting (Fig. 1b), and multi-channel retroreflection (Fig. 1c).

As depicted in Fig. 1d left, the incident wave, which is illuminated into a gradient metasurface, will be reflected abnormally, resulting from abrupt phase changes over the scale of the wavelength. Then, the abnormal reflective angle can be obtained readily according to the generalized Snell’s law. In the case of the periodic artificial structure, multi-harmonics can be generated and reflected in multiple directions when the incident wave impinges on it, as shown in Fig. 1d left. However, the periodic artificial structure generally refers to gratings, photonic crystals, etc, which have limited control freedom, while the gradient metasurface has rich control freedom, such as size and rotation. Therefore, it is crucial to introduce periodic properties into phase gradient metasurfaces for the effective manipulation of the multi-harmonic. Here, the SH-GSL with periodic metasurfaces is presented.

With consideration of the effect of phase wrapping, the gradient phase metasurface can also be regarded as a periodic structure. The meta-atoms within a period form a supercell. Then, the periodic supercell induces the effect of spatial harmonics. The propagating wave vectors of spatial harmonics, ignoring losses, can be described according to the Floquet period theory as follows:

3 $${{k}}_{\ell }={{k}}_{0}\,\sin \,{\theta }_{i}+\frac{2\pi \ell }{Np},\,\ell \in Z$$

where *k*_*ℓ*_ represents the wave vector of the reflected wave due to the period of the metasurface and *ℓ* denotes the order of the reflective harmonic waves. Besides, *N* and *p* are the number of meta-atoms in each period and the size of the meta-atom, respectively. According to (3), each order of spatial harmonics can be modulated by the periodic metasurface theoretically.

The abnormal reflection of metasurfaces can be analyzed sufficiently by extending the generalized Snell’s law with period properties. Generally, the phase gradient Δ*k*_*∥*_ = ΔΦ/Δ*x* in metasurface design is based on the fundamental harmonic (*ℓ* = 0) and is therefore independent of the harmonic order *ℓ*. This phase gradient is determined solely by the geometric arrangement and material properties of the meta-atoms. Due to the periodicity of the metasurface, the scattered fields satisfy the Floquet theorem, where the *ℓ*-th harmonic order introduces an additional momentum compensation of 2*πℓ*/*Np*. This results in an equivalent phase gradient for the *ℓ*-th order:

4 $$\varDelta {k}_{//}^{\ell }=\varDelta {k}_{//}+\frac{2\pi \ell }{Np},\,\ell \in Z$$

where Δ*k*_*∥*_ = ΔΦ/Δ*x* represents the phase gradient designed based on the fundamental harmonic, and $\varDelta {k}_{//}^{\ell }$ represents the equivalent phase gradient for the *ℓ*-th spatial harmonic. Accordingly, the fundamental harmonic with *ℓ* = 0 and all the other-order harmonics with *ℓ* ≠ 0 can be simultaneously manipulated to achieve individual radiation or non-radiation.

Furthermore, assume that each supercell of the metasurface comprises *N* meta-atoms with the phase difference between adjacent meta-atoms of ΔΦ. The intrinsic wave vector for compensation in the transverse direction is written as:

5 $${\boldsymbol{\Delta }}{{\bf{k}}}_{//}={{\bf{e}}}_{{\boldsymbol{x}}}\frac{\varDelta \varPhi }{\varDelta x}={{\bf{e}}}_{{\boldsymbol{x}}}\frac{1}{p}[\varPhi (x+p,y)-\varPhi (x,y)]$$

where **e**_***x***_ is the unit direction vector parallel to the metasurface, and the direction of the wave vector compensation is along the *x*-direction. For a metasurface designed with the linear phase progression assumption, the inter-unit phase differences are constant (ΔΦ/Δ*x* = Δ*φ*/*p*, Δ*φ* = constant), leading to a simplified expression: ΔΦ/Δ*x* = (*N*Δ*φ*)/(*Np*) = [Φ(*x* + *Np*, *y*) – Φ(*x*, *y*)]/*Np*.

According to (4), the total compensation wave vector is related to the effect of the phase difference between adjacent meta-atoms and the periodic characteristics of the overall supercell, taking into account the proposed theory as:

6 $${\boldsymbol{\Delta }}{{\bf{k}}}_{//}^{{\bf{t}}{\bf{o}}{\bf{t}}{\bf{a}}{\bf{l}}}={{\bf{e}}}_{{\boldsymbol{x}}}=\left(\frac{\varDelta \varPhi }{\varDelta x}+\frac{2\ell \pi }{Np}\right){\boldsymbol{\Delta }}{{\bf{k}}}_{//}+{{\bf{e}}}_{{\boldsymbol{x}}}\left(\frac{2\ell \pi }{Np}\right),\,\ell \in Z$$

In general, ΔΦ = 2*π* is selected as the compensation phase of the supercell of the metasurface, i.e., ΔΦ/(*Np*) = 2*π*/(*Np*). The metasurface introduces a transverse momentum compensation through its periodic architecture while maintaining conservation of the total wavevector magnitude (| **k** | = *k*_0_) in free space. Concurrently, Fresnel reflection at the dielectric interface induces a *π*-phase reversal (ΔΦ = *π*) when electromagnetic waves interact with a medium of higher refractive index. By integrating the transverse wavevector conservation principle (governed by the momentum conservation) and incorporating the *π*-phase shift (commonly referred to as the half-wave loss condition in classical optics), the governing wave-vector equation is derived as:

7 $${\boldsymbol{\Delta }}{{\bf{k}}}_{//}^{{\bf{t}}{\bf{o}}{\bf{t}}{\bf{a}}{\bf{l}}}+{{\bf{k}}}_{{\bf{i}}}+\boldsymbol{\mathscr{K}}({{\bf{k}}}_{{\bf{i}}})={{\bf{k}}}_{{\bf{r}}}$$

8 $${{\bf{k}}}_{{\bf{i}}}={{\bf{e}}}_{{\boldsymbol{x}}}{n}_{i}{k}_{0}\,\sin \,{\theta }_{i}+{{\bf{e}}}_{{\boldsymbol{z}}}{n}_{i}{k}_{0}\,\cos \,{\theta }_{i}$$

9 $${{\bf{k}}}_{{\bf{r}}}={{\bf{e}}}_{{\boldsymbol{x}}}{n}_{i}{k}_{0}\,\sin \,{\theta }_{r}+{{\bf{e}}}_{{\boldsymbol{z}}}{n}_{i}{k}_{0}\,\cos \,{\theta }_{i}$$

where $\boldsymbol{\mathscr{K}}({\bf{k}}{\bf{i}})$ represents the item of half-wave loss with the expression as follows:

10 $$\boldsymbol{\mathscr{K}}({{\bf{k}}}_{{\bf{i}}})=-2{{\bf{e}}}_{z}({{\bf{k}}}_{{\bf{i}}}\cdot {{\bf{e}}}_{z})$$

These formulations rigorously satisfy both momentum conservation in the transverse plane and the boundary-matching condition for phase discontinuities.

It should be noted that the time-harmonic dependence is denoted as *e*^*jωt*^ in this paper.

Since the metasurface introduces a transverse momentum compensation Δ*k*_*∥*_, the longitudinal wavevector component *k*_*z*_ is determined by momentum conservation ${k}_{z}=\sqrt{{k}_{0}^{2}-{k}_{x}^{2}}$. Consequently, the transverse terms in (7) can be expressed in scalar form as:

11 $${n}_{i}{k}_{0}\,\sin \,{\theta }_{r}-{n}_{i}{k}_{0}\,\sin \,{\theta }_{i}=\varDelta {k}_{//}+\frac{2\ell \pi }{Np}$$

which can be degraded as the generalized Snell’s law in the case of *ℓ* = 0.

Similarly, for refraction, it can be derived as the following expression:

12 $${n}_{t}{k}_{0}\,\sin \,{\theta }_{t}-{n}_{i}{k}_{0}\,\sin \,{\theta }_{i}=\varDelta {k}_{//}+\frac{2\ell \pi }{Np}$$

where *θ*_*t*_ is the angle of refraction and *n*_*t*_ is the refractive index of the medium on the other side. The refraction validation can be seen in Supplementary Material S10.

The SH-GSL framework enables selective radiative harmonic excitation while suppressing non-radiative modes to the near-field regime (Fig. 1d middle). This selectivity unlocks advanced wavefront engineering capabilities—including abnormal-harmonic reflection/refraction, multi-beam splitting, and multi-channel retroreflection—surpassing the limitations of conventional approaches. In addition, the framework reveals a persistent harmonic (termed the mirror mode) governed by intrinsic momentum conservation, which resists metasurface manipulation and strictly adheres to the conventional Snell’s law. Rather than a limitation, this invariant behavior provides a foundational symmetry for designing multifunctional devices: deliberate retention of the mirror mode enables complex multi-channel operations within the same metasurface platform (multi-channel retroreflection in Fig. 1d right).

**Fig. 2:**
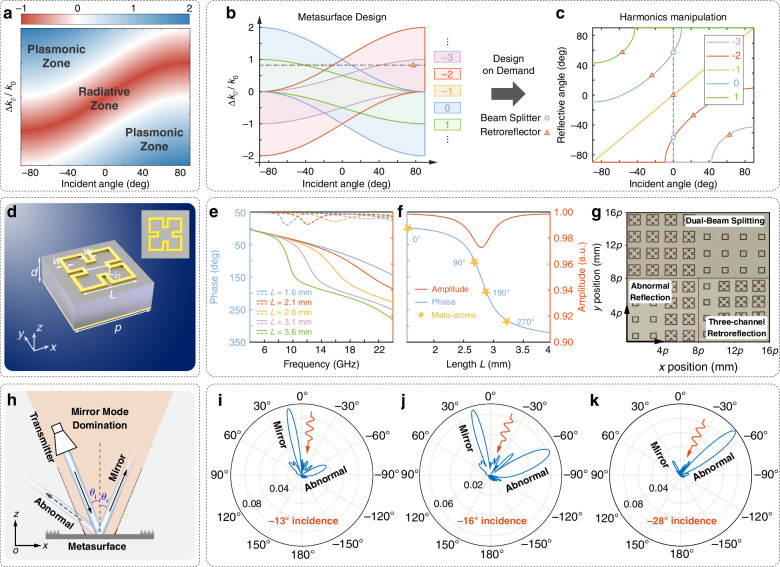
The analysis of SH-GSL, designed meta-atoms, and the mirror mode. **a** The schematic of the deterministic Floquet-engineered momentum compensation mechanism. By engineering the incident angle and compensation wave vector, each spatial harmonic can be confined in the plasmonic zone or radiative zone, thereby enabling different manipulations. **b** The relationship of the incident angle and the compensation wave vector. Different regions represent the radiative zones of different harmonics, and out-of-region means that the harmonic is localized on the surface. **c** The relationship of incident and reflective angles of different harmonics after designing the compensation wave vector (0.8*k*_0_) of the metasurface. **d** The schematic of the meta-atom. **e** The amplitude and phase response of the meta-atom from 4 GHz to 24 GHz. **f** The amplitude and phase response of the meta-atom from *L* = 1.6 mm to *L* = 4.0 mm at 14 GHz. **g** The schematic of the supercell of the metasurfaces: dual-beam splitting, abnormal harmonics reflection, and three-channel retroreflection. **h** The illustration of the dominant area of the mirror mode, where the abnormal reflection is much lower than the mirror reflection. The analysis of different far-field patterns under **i** −13°, **j** −16°, and **k** −28° incidence, which represent the cases of dominant mirror reflection, mirror equal to abnormal reflection, and dominant abnormal reflection, respectively

According to (2) and (11), the wave vector of the reflected wave is the sum of the wave vector of the incident wave and the compensation wave vector of the metasurface, i.e., $\varDelta {k}_{//}^{r}$ = *k*_i_sin*θ*_i_ + (Δ*k*_*∥*_ + 2*ℓπ*/*Np*). Therefore, each harmonic corresponds to a reflected wave vector. The state of each harmonic will change in the radiative zone and the plasmonic zone with the variation of the incident angle and the compensation wave vector of the metasurface (Fig. 2a): When $|\varDelta {k}_{//}^{\ell }|/{k}_{0}\le 1$, the state of the harmonics is manifested as a radiative state. Otherwise, the harmonics will be confined as plasmonic waves in the near field. Notably, in Fig. 2a, color indicates |Δ*k*_*∥*_ + 2*πℓ*/*Np* | /*k*_0_ – 1, dimensionless; positive values denote radiative zones, negative values denote plasmonic zones. The *y*-axis unit is ‘1’ since it represents the ratio. The asymmetry of the color range for *ℓ* ≠ 0 arises from the offset Δ*k*_*∥*_ + 2*πℓ*/*Np*. The relationship between each harmonic, the incident angle, and the compensation wave vector is illustrated in Fig. 2b, and the colored areas represent the radiative zones of each harmonic. It is worth noting that, generally due to phase wrapping, the phase coverage of a metasurface over one period is ΔΦ = 2*π*, i.e., Δ*k*_*∥*_ = 2*π*/*Np*. Therefore, when the order of the spatial harmonic is *ℓ* = −1, the condition of radiation $|\varDelta {k}_{//}^{r}|/{k}_{0}\le 1$ can always be satisfied, meaning that the −1st spatial harmonic will always exist in the radiation zone. In this case, the phase gradient introduced by the metasurface cancels out with the periodic characteristic of the metasurface, and the manipulation of electromagnetic waves by the entire metasurface conforms to the traditional Snell’s law, denoted as the mirror mode (i.e., *k*_i_sin*θ*_i_ = *k*_r_sin*θ*_r_). Therefore, the −1st order spatial harmonic cannot be manipulated utilizing the periodic gradient metasurface.

The metasurface design strategy follows distinct parameter-selection criteria depending on functional purposes:

1. Single-beam manipulation: Achieved by selecting incident angles and compensating wavevectors within non-overlapping colored zones (Fig. 2b), ensuring phase-gradient isolation for unidirectional wavefront control;
2. Multi-beam operation: Requires parameters from overlapping colored zones, where coordinated phase discontinuities enable simultaneous multi-channel wavefront shaping;
3. Plasmonic excitation: Parameters external to these zones prioritize near-field momentum matching, yielding high-efficiency surface wave generation while suppressing far-field radiation.

For example, at a compensation wave vector of Δ*k*_*∥*_ = 0.8*k*_0_ (dash line in Fig. 2b), the SH-GSL framework generates five radiative spatial harmonics (*ℓ* = −3 to +1) spanning the incident space (Fig. 2c). This harmonic diversity enables simultaneous multifunctional operation, such as a dual-beam splitter and a five-channel retroreflector, within a single metasurface platform. Such multi-channel control—seen as a parasitic interference in conventional phase-gradient metasurfaces—demonstrates the framework’s capacity to harness harmonic multiplicity for unprecedented wavefront engineering.

### Analysis and design of meta-atoms

By spatially arranging meta-atoms within the metasurface, one can design the phase gradient along the interface of the metasurface, tailoring the wavefront of the reflected and refracted waves. To construct metasurfaces to validate the proposed theory, we used the Minkowski loop structure as the meta-atom, which offers excellent geometric symmetry, reducing its size while broadening the operational bandwidth^49^. As shown in Fig. 2d, the Minkowski loop is placed on a substrate with a thickness of 1.52 mm (0.07*λ*) and a relative permittivity of 3.48 − 0.013i. A metallic plate on the backside of the meta-atom enhances reflectivity. The period of the meta-atom (denoted as *p*) is set to 4.25 mm. This single-layer metasurface achieves the desired phase distribution by adjusting the propagation phases. Additionally, the phase gradients can be varied by altering the structural parameter, *L*, of the Minkowski loop, as demonstrated in Fig. 2e. This figure shows the amplitude and phase response to linearly polarized incidence, with reflectivity near 1.0 for *L* values ranging from 1.6 mm to 3.6 mm. Notably, though the operating frequency of the metasurfaces is set at 14 GHz, the meta-atom also provides a phase coverage of over 2*π* at frequencies greater than 14 GHz.

At the operating frequency of 14 GHz, the reflectivity exceeds 0.97, and the phase distribution covers nearly 2*π* for *L* values between 1.6 mm and 4.0 mm (Fig. 2f). These characteristics meet the requirements for the reflective metasurface design. It is important to note that a full 2*π* coverage of the reflective phase is not strictly necessary^38^. Based on parameter scanning, four meta-atoms (0°, 90°, 180°, 270°) are selected to construct different metasurfaces—dual-beam splitting and abnormal-harmonic reflection with two meta-atoms of 0° and 180°, and three-channel retroreflection with the four meta-atoms (Fig. 2g). The selected meta-atoms have *L* values of 1.60 mm, 2.70 mm, 2.89 mm, and 3.17 mm with detailed structural parameters listed in Table 1.

**Table 1:** Structure parameters of the meta-atom

Parameter letter	Parameter size	Unit name
*p*	4.25	mm
*d*	1.52	mm
*L*	Parameter sweep	mm
*w*	0.20	mm
*L*_in_	1.50	mm

Notably, as mentioned above, the mirror reflection mode associated with *ℓ* = −1 always exists. Once the metasurface design is completed, it is essential to evaluate the proportion of the mirror mode compared to other spatial harmonics, i.e., to determine whether the mirror mode is the prevailing one. Taking the metasurface that uses three spatial harmonics (*ℓ* + 1 = 0, ±1) as an example, the spatial harmonics, except for the mirror mode (*ℓ* + 1 = 0), should not exist in free space. Thereby, by confining other spatial harmonics in the plasmonic zone, it can be derived as follows:

13 $$|{k}_{0}\,\sin \,{\theta }_{r}|=|\left(\varDelta {k}_{//}+\frac{2\ell \pi }{Np}\right)+{k}_{0}\,\sin \,{\theta }_{i}|\ge {k}_{0},\,\ell +1=\pm 1$$

According to the different designs of the metasurface (especially the compensation wave vector and the supercell), the corresponding range of incident angle can be calculated. Within this incident range, the reflected wave is mainly manifested as the mirror reflection, while the abnormal reflection is negligible (Fig. 2h). This implies that the compensating wave vector Δ*k*_*∥*_ of the metasurface is already known and fixed, and it is necessary to calculate a specific range of incident angles within which the mirror mode prevails. Furthermore, the influence of the mirror mode beyond the specified range can be effectively considered negligible. Detailed analysis can be seen in Supplementary Material S2.

Taking Δ*k*_*∥*_ = 1.26*k*_0_ as an example, simulations are conducted using COMSOL 6.2, and the mirror mode is calculated to dominate in the range of ~−15.1° to 15.1° according to (13). To explore different cases of incidence, simulations were performed for three incidence angles of −13°, −16°, and −28°, with far-field patterns shown in Fig. 2i–k, respectively. At the incident angle of −13° (within the mirror mode domination, Fig. 2i), the mirror reflection significantly exceeds the abnormal reflection. At the incident angle of −16° (approximately the angle threshold, Fig. 2j), the mirror and abnormal reflections are nearly equal, marking the boundary of the mirror mode region. For the incident angle of −28° (beyond the mirror mode domination, Fig. 2k), the abnormal reflection dominates, indicating a departure from the mirror mode region. The energy distribution analysis^50^ of the mirror mode and other spatial harmonics can be seen in Supplementary Material S1.

### Applications based on SH-GSL

We demonstrate the SH-GSL framework through three functional metasurfaces—abnormal spatial-harmonic reflection, multi-beam splitting, and multi-channel retroreflection—constructed using periodically arranged meta-atoms (Fig. 2g). Note that enhanced spatial harmonic generation emerges when supercell dimensions approach the operational wavelength, and we propose the design of repeating-cell metasurface architectures to further amplify high-order harmonics through engineered phase gradients. For example, we can construct a metasurface with a phase gradient of 45° using eight meta-atoms (0, *π*/4, *π*/2, *3*π/4, *π*, 5*π*/4, 3*π*/2, and 7*π*/4), whose period is much smaller than the wavelength. We can design such a metasurface using a repeating-cell metasurface composed of four repeating ‘0’ and four repeating ‘*π*’ meta-atoms, which has a period closer to the wavelength, thereby exhibiting stronger harmonic generation capabilities. Scalable supercell expansion, achieved by replicating base meta-atom configurations (constructing a larger period), enables precise multi-channel wavefront control while maintaining fabrication simplicity. This approach establishes a generalized design paradigm for multifunctional meta-devices requiring the manipulation of multiple spatial harmonics.

#### SH-GSL engineered abnormal spatial-harmonic reflection

**Fig. 3:**
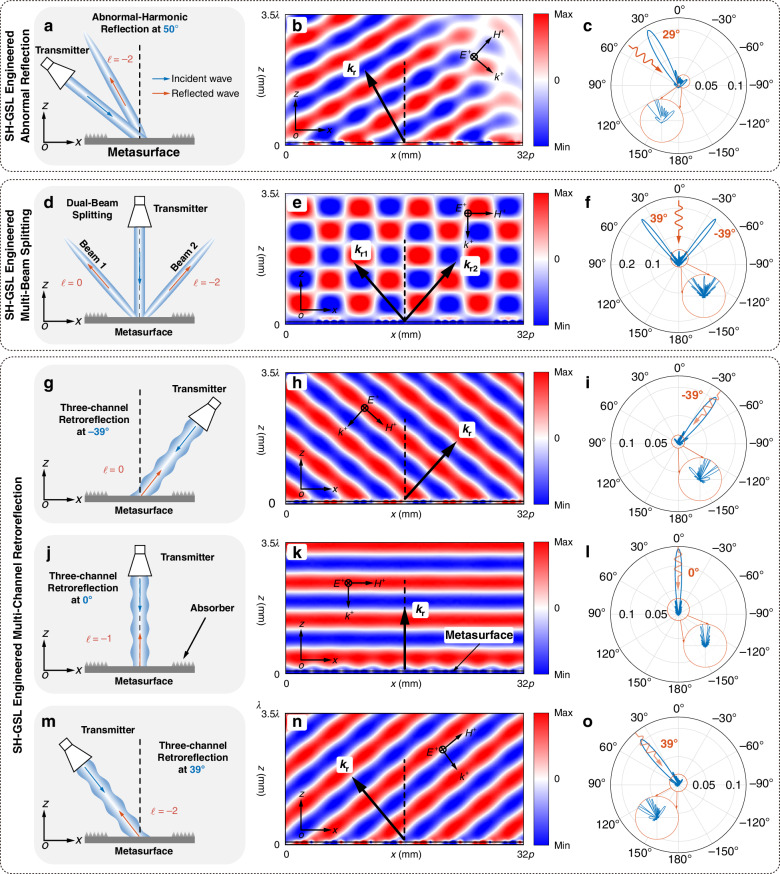
The simulation results of the abnormal-harmonic reflection, beam splitting, and multi-channel retroreflection. **a** The schematic of the simulation for abnormal-harmonic reflection at 50° incidence. **b** The distributions of the electric field in the *xoz* plane at 50°. **c** The far-field pattern of the abnormal-harmonic reflection at 50°. **d** The schematic of simulations for dual-beam splitting. The incident wave is illuminated vertically onto the metasurface, and the reflected wave is divided into two beams radiating into free space at 39° (0th harmonic) and –39° (−2nd harmonic), respectively. **e** The distributions of the electric field in the *xoz* plane. **f** The far-field pattern of the dual-beam splitting. The schematic of simulations for three retroreflective channels: **g** 0th harmonic corresponding to −39°, **j** −1st harmonic corresponding to 0°, and **m** −2nd harmonic corresponding to 39°. The incident plane waves illuminate the metasurface at three retroreflective angles, and the reflected waves are reflected along the same direction. The distributions of the electric field at **h** −39°, **k** 0°, and **n** 39° incidence in the *xoz* plane. The distributions are all relative electric fields, and the metasurface is placed on the bottom. The far-field patterns at **i** −39°, **l** 0°, and **o** 39° incidence in the *xoz* plane

We design a prototype metasurface comprising periodically arranged ‘0’ and ‘*π*’ phase meta-atoms (Fig. 1a). Each supercell integrates two ‘0’ and two ‘*π*’ meta-atoms with period *P* = 4*p* (*N* = 4 meta-atoms, each of period *p*). Two same meta-atoms construct a new meta-atom with a larger period of 2*p* (2 + 2 repeating supercells). In this case, the phase gradient of the adjacent larger meta-atoms changes to *π*. In our work, we define the phase gradient ΔΦ relative to the 0th-order spatial harmonic; all other spatial harmonics inherit their effective phase gradient (including its sign) based on this ΔΦ. To uniquely determine each spatial harmonic order, we explicitly set the positive phase gradient direction along the +*x* direction. Under this convention, the SH‑GSL formalism can be applied unambiguously to assign each spatial harmonic and its effective phase gradient. Analogously, we can use this method to create a repeating-cell metasurface composed of the two kinds of meta-atoms (‘0’ and ‘*π*’) to form an arbitrary compensation wave vector. The SH-GSL framework predicts three radiative spatial harmonics (*ℓ* = −2–0) across the incident space. We calculate and simulate the reflection angles for each harmonic using SH-GSL (Fig. S1b), demonstrating precise angular dispersion control through harmonic-specific phase engineering (the simulation results at the incidence of 50° are illustrated in Fig. 3a–c). Besides, the mirror mode domination can be illustrated in Fig. S1b.

#### SH-GSL engineered multi-beam splitting

A beam splitter is a common device for dividing an incident beam into two or more separate beams. We demonstrate multi-beam splitting in the same incident plane through harmonic engineering in metasurfaces under normal illumination. By systematically tuning the compensation wave vector, multiple spatial harmonics (*ℓ* = −3 to +1, except for *ℓ* = −1) are selectively activated via the SH-GSL framework (Fig. 2b). Two distinct configurations are achieved and simulated: Dual-beam splitting ( ± 39°) using *ℓ* = 0 and −2 spatial harmonics through 4 + 4 repeating supercells of alternating ‘0’/‘*π*’ meta-atoms (Fig. 1a, simulation results in Fig. 3d–f); Quad-beam splitting (−57.2°, −24.8°, +24.8°, +57.2°) via *ℓ* = −3, −2, 0, +1 spatial harmonics in 6 + 6 repeating supercells of alternating ‘0’/‘*π*’ meta-atoms (Fig. S2). The energy distribution of spatial harmonics can be seen in Supplementary Material S1.

This harmonic-selective design establishes a direct correspondence between meta-atom arrangements (binary ‘0’/‘*π*’ phase elements) and angular dispersion characteristics, enabling predictable multi-channel wavefront manipulation beyond conventional single-beam metasurfaces.

#### SH-GSL engineered multi-channel retroreflection

Retroreflectors are devices that can reflect electromagnetic waves along their incident direction with minimal scattering losses^31,36,40^. Utilizing the manipulation of multiple spatial harmonics, the multi-channel retroreflector can be achieved based on single-layer metasurfaces, which cannot be explained by GSL. With the condition of the retroreflection *θ*_i_ = −*θ*_r_, the relationship of an *m*-channel retroreflector among retroreflective angles, compensation wave vector, and spatial harmonics can be derived as follows:

14 $$\begin{array}{lll}{\theta }_{i}(\ell )=\left\{\begin{array}{ll}0, & \ell +1=0\\ \mp \arcsin (\frac{\Delta {k}_{//}}{2{k}_{0}}), & \ell +1=\pm 1\\ \vdots & \vdots \\ \mp \arcsin [\frac{(m-1)\Delta {k}_{//}}{4{k}_{0}}], & \ell +1=\pm \frac{m-1}{2}\end{array}\right.\end{array}$$

where

15 $$|\varDelta {k}_{//}|\in \left[\frac{4{k}_{0}}{m+1},\frac{4{k}_{0}}{m-1}\right)$$

Detailed analysis can be seen in Supplementary Materials S5. In this way, the incident (retroreflective) angles, the corresponding retroreflective channels, and the harmonics of the *m*-channel retroreflector can be calculated using (14) (Fig. 1d right). Simultaneously, the compensation wave vector of the *m*-channel retroreflector can be selected and designed according to (15).

Notably, while substantial research efforts have been devoted to investigating geometric-phase-enabled retroreflectors through GSL implementations, these established paradigms remain persistently constrained to single-channel retroreflection analyses. In striking contrast, our proposed theory fundamentally advances this field by establishing a systematic framework that accounts for all multichannel retroreflection characteristics in metasurfaces utilizing spatial harmonics.

Furthermore, the design purpose of the ‘perfect’ retroreflector is presented: for each channel of the multi-channel retroreflector, only the spatial harmonic, which the specific retroreflective mode corresponds to, exists in this channel to generate pure retroreflections. This means that the other undesired spatial harmonics do not exist in the radiation field or are sufficiently suppressed around the reactive near field. By designing the retroreflective angle and the compensation wave vector, the state of spatial harmonics will change in the radiative zone and the plasmonic zone (Fig. 2a). Assume that retroreflection occurs in the case of the incident angle of *θ*_i_, the order of the corresponding spatial harmonic is *ℓ*_retro_, and the rest orders are *ℓ*_other_. In this case, the retroreflective angle can be denoted as *θ*_i_(*ℓ*_retro_) according to (14). Then, the perfect retroreflection can be achieved by suppressing undesired spatial harmonics as surface plasmons around the reactive near field. This means that ∀ *ℓ* ∈ {*ℓ* ≠ *ℓ*_retro_} ∩ {*ℓ* ≠ − 1}, the following expression holds:

16 $$|{k}_{0}\,\sin \,{\theta }_{r}|=|(\ell +1)\Delta {k}_{//}+{k}_{0}\,\sin \,{\theta }_{i}|\ge {k}_{0}$$

To better describe the case of the perfect multi-channel retroreflection, the method of Logical Multiplication is adopted here. Assuming that event *A* occurs under condition *B*, it can be expressed as *P*(*A* | *B*) = 1, otherwise *P*(*A* | *B*) = 0. Therefore, when event *A* occurs under all conditions *B*, *C*, and *D*, it can be expressed as

17 $$P(A|B)\cdot P(A|C)\cdot P(A|D)=1$$

where once the event *A* is invalid under any condition, the value of (17) is equal to 0.

Accordingly, the expression in (16) can be defined as event *A*, and condition *B* is *ℓ* ∈ {*ℓ* ≠ *ℓ*_retro_} ∩ {*ℓ* ≠ − 1}, i.e.,

18 $$\left\{\begin{array}{l}A=\{|(\ell +1)\varDelta {k}_{//}+{k}_{0}\,\sin [{\theta }_{i}({\ell }_{retro})]|\ge {k}_{0}\}\\ B=\{\ell \ne {\ell }_{\mathrm{retro}}\wedge \ell \,\ne \,-1\}\end{array}\right.$$

where *ℓ* ∈ Z, and the condition {*ℓ* ≠ −1} is due to the mirror-reflection mode that always exists. Furthermore, *P*(*A* | *B*) expressed by (18) could represent whether the case of the retroreflection corresponding to *ℓ*_retro_ is perfect or not. Note that, *P*(*A* | *B*) = 1 indicates that for any arbitrary *ℓ* except for *ℓ*_retro_, the inequality in the event *A* holds, i.e., the case of the retroreflection corresponding to *ℓ*_retro_ is perfect; 0 < *P*(*A* | *B*) < 1 represents that there exists at least one spatial harmonic *ℓ* in the radiation field resulting in the imperfect retroreflection; *P*(*A* | *B*) = 0 signifies that all spatial harmonics *ℓ* are radiated into free space, which leads to the imperfect retroreflection.

In addition, since each incident angle (retroreflective angle) *θ*_i_ corresponds to the specific *ℓ*-th spatial harmonic, the order of spatial harmonic can be expressed as a function of the incident angle, i.e., *ℓ*(*θ*_i_). As for the perfect retroreflector, at the incident angle *θ*_i_, only *ℓ*(*θ*_i_)-th harmonic can be retroreflected, and others are suppressed as surface plasmons. Therefore, the range of the compensation wave vector of the perfect all-channel (*m*-channel) retroreflector needs to be satisfied according to (14) as:

19 $$\mathop{\prod }\limits_{{\theta }_{i}^{(n)}={\theta }_{i}^{(1)}}^{{\theta }_{i}^{(m)}}{P[{A}_{n}({\theta }_{i}^{(n)})|{B}_{n}({\theta }_{i}^{(n)})]|}_{{\theta }_{i}^{(n)}={\theta }_{i}({\ell }_{\mathrm{retro}}^{(n)})}=1,\,{\ell }_{\mathrm{retro}}^{(n)}+1\in \left\{0,\,\pm 1,\,\mathrm{..}.,\,\pm \frac{m-1}{2}\right\}$$

where ${\theta }_{i}^{(n)}={\theta }_{i}({\ell }_{retro}^{(n)})$ can be calculated according to (14). In summary, the compensation wave vector of the perfect *m*-channel retroreflector must be satisfied as:

20 $$|\varDelta {k}_{//}|\in [{k}_{0},\infty )$$

Detailed discussion can be seen in Supplementary Materials S5. Notably, for the specific three-channel design, the additional requirement |Δ*k*_*∥*_|∈[*k*_0_, 2*k*_0_) guarantees that exactly one harmonic is radiative at each of the three incidence angles and all others are evanescent. Combining with the general perfect *m*-channel case |Δ*k*_*∥*_|∈[*k*_0_, ∞)—and assuming lossless elements and an infinite-array approximation—the incident energy in each channel is fully redirected back with no parasitic scattering, making the three-channel retroreflector inherently “perfect.” Therefore, only single and three-channel retroreflectors can achieve perfect retroreflection, whereas a retroreflector featuring more than three channels cannot achieve perfect retroreflection.

A perfect three-channel retroreflector (Fig. 1c) and a five-channel retroreflector (Fig. S5a) are designed and simulated in this paper. In this case, we select four meta-atoms with a phase gradient of *π*/2 to construct the supercell without the need to use the repeating-cell metasurface. In addition, the metasurface used for the abnormal-harmonic reflection is also a three-channel reflector. The five-channel retroreflector still uses the form of repeating-cell metasurfaces (3 + 3 repeating supercells of alternating ‘0’/‘*π*’ meta-atoms, as shown in Fig. S5a) to enhance the generation of spatial harmonics. The results of the three-channel retroreflector and the five-channel retroreflector are illustrated in Fig. 3g–o and Fig. S5, respectively. From the far-field pattern, the retroreflection of the three-channel retroreflector is cleaner, without interference from other spatial harmonics due to its ‘perfect’ property, while the five-channel retroreflector is affected by interference from other spatial harmonics.

### Results of experiments

In this paper, a single horn is used to both transmit and receive electromagnetic waves, providing far-field radiation, which is different from the conventional approach that uses two horns to calculate the far-field pattern. To theoretically analyze the far-field radiation of the retroreflector, the method for calculating the far-field radiation is adopted as follows:

21 $${S}_{r}^{\text{real}}=D({\theta }_{{\rm{i}}}-{\theta }_{\text{retro}})\cdot \,\cos |{\theta }_{{\rm{i}}}+{\theta }_{{\rm{r}}}|$$

where ${S}_{r}^{\text{real}}$ is the real receiving signal, representing the far-field radiation with a single horn; *D*(*θ*) is the directivity coefficient of the receiving antenna, as described as follows:

22 $$D(\theta )=\frac{1+\,\cos \,\theta }{2}\cdot \frac{\sin ({k}_{0}a/2\,\sin \,\theta )}{{k}_{0}a/2\,\sin \,\theta }$$

where *a* is the aperture of the receiving antenna. (Detailed analysis can be seen in Supplementary Materials S7).

**Fig. 4:**
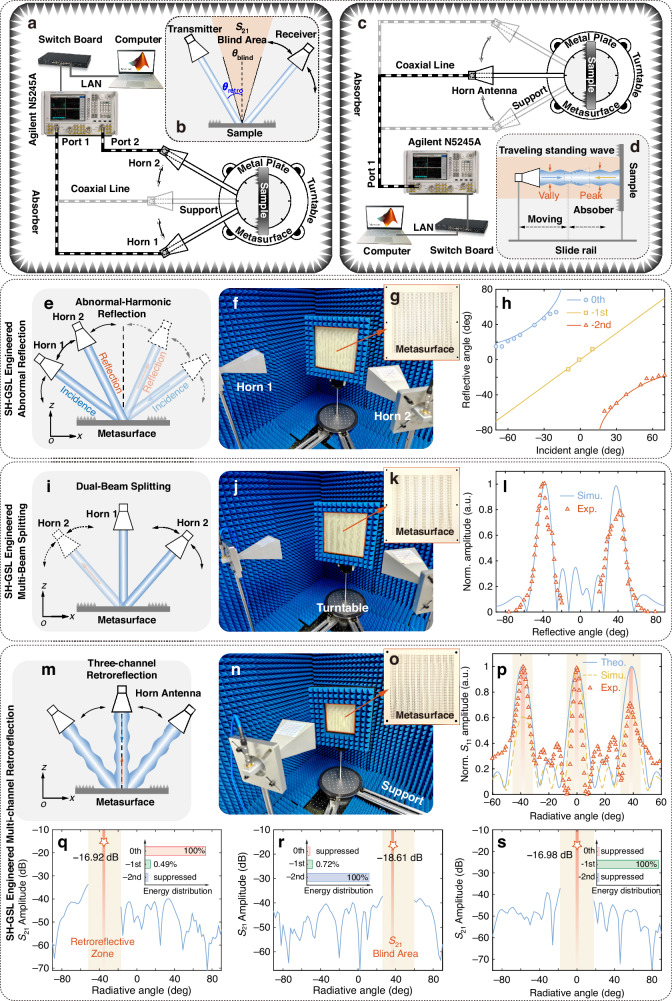
Schematic diagram of the experiments and experimental results. **a** The schematic of the dual static experiment for *S*_21_. The metasurface or the metal plate is placed on the turntable, and the two horns are used to obtain *S*_21_. **b** The illustration of the blind area between two horns due to the support arms. **c** The schematic of the single static experiment for *S*_11_. The metasurface or the metal plate is placed on the turntable, and the single horn is used to obtain *S*_11_. **d** The illustration of the traveling standing wave resulting from the highly efficient retroreflection. The schematics of the measurement of the abnormal-harmonic reflection (**e**), dual-beam splitting (**i**), and three-channel retroreflection (**m**). The photos of the actual experimental scenes (**f** the abnormal-harmonic reflection, **j** dual-beam splitting, and **n** three-channel retroreflection). The fabricated metasurfaces for the abnormal-harmonic reflection (**g**), dual-beam splitting (**k**), and three-channel retroreflection (**o**). **h** The comparison of results for the abnormal harmonics reflection (solid lines represent theoretical results, and the circle, square, and triangle represent experimental results). **l** The comparing results for the beam splitter (solid lines represent theoretical results, and the triangle represents experimental results). **p** The comparing results for the three-channel retroreflector (solid lines represent theoretical results, dashed lines represent simulation results, and the triangle represents experimental results). In retroreflection, the energy distributions of different spatial harmonics under **d** −39°, **e** 39°, and **f** 0° incidence, respectively. The results of the far-field radiation under **q** −39°, **r** 39°, and **s** 0° incidence, and the inserts represent the energy distributions of different spatial harmonics (0th, −1st, −2nd), respectively

The metasurfaces mentioned above are fabricated using Printed Circuit Board (PCB) technology (Fig. 4g: 56 × 56 meta-atoms for abnormal harmonics reflection, Fig. 4k: 56 × 56 meta-atoms for dual-beam splitter, and Fig. 4o: 40 × 40 meta-atoms for three-channel retroreflector).

#### SH-GSL engineered abnormal spatial-harmonic reflection

We implement two experimental configurations (Fig. 4a, c) to overcome angular measurement constraints imposed by the turntable’s blind area (Fig. 4b). The metasurface is fixed on the vertical back plate of a turntable, with non-metasurface areas covered in absorbing material. The experiment is conducted in an anechoic chamber to minimize electromagnetic interference. The first single static configuration measures reflection coefficients (*S*_11_), while the second dual static captures transmission-derived far-field patterns (*S*_21_) (Fig. 4e), where *S*_21_ maxima correspond to dominant harmonic reflection angles (Fig. S3). To resolve retroreflection obscured by the blind area, we combine the angular data of *S*_21_ with *S*_11_ (Fig. 4h). Due to efficient retroreflection, a standing wave forms between the transmitting horn and the metasurface, as depicted in Fig. 4d. To ensure maximum reception, the transmitting horn is positioned at the peak of the standing wave.

The metasurface exhibits three radiative spatial harmonics (*ℓ* = −2, −1, and 0), with experimental reflection angles (symbols: rectangles, squares, and circles) showing small deviation ( < 5°) from numerical simulations (solid lines). This agreement validates the SH-GSL framework’s predictive accuracy for harmonic-specific angular dispersion. Notably, the observed mirror-mode dominance range (−16° to +6°, approximately) aligns with theoretical analysis (−15.1° to +15.1°). Minor discrepancies between experimental and simulated results arise from fabrication tolerances in meta-atom dimensions and experimental uncertainties in angular alignment. We compute conversion efficiencies by normalizing the anomalous-reflected power in each harmonic channel to the reference metal reflection under normal incidence (*S*_11_ = − 13.8 dB).

The maximum efficiency for each harmonic occurs at its design incident angle and declines for off-design angles, as shown in Table 2. This confirms that the metasurface not only directs the reflected beam to the intended angles but also does so with high efficiency at those angles (reflection efficiency >50% within ±55°), and the reflection efficiency is correlated with the proportion of harmonic energy.

**Table 2:** The maximum efficiency for each harmonic

Incident angle	−70°	−65°	−60°	−55°	−50°	−40°	−30°
Efficiency	11.48%	24.43%	38.90%	58.88%	77.62%	retro. range	79.42%
Incident angle	−25°	−20°	−10°	0°	10°	20°	25°
Efficiency	64.56%	45.70%	47.86%	retro. range	52.48%	61.65%	72.44%
Incident angle	30°	40°	50°	55°	60°	65°	70°
Efficiency	79.43%	retro. range	74.13%	69.18%	58.21%	36.39%	22.90%

#### SH-GSL engineered dual-beam splitting

We characterize the metasurface’s far-field response using a dual-horn antenna configuration (Fig. 4c): one antenna fixed at 0° illuminates normally the sample, while the receiver rotates from −80° to +80° to map angular scattering (Fig. 4i). Figure 4l compares simulated (solid blue curve) and experimental (discrete red squares) angular dispersion, revealing weaker mirror reflection at 0° and close agreement with small deviation (<3°) in beam-splitting angles (measured: 39°, 41°; simulated: 39° ± 0.5°). This quantitative alignment validates the SH-GSL framework’s predictive accuracy for harmonic-driven beam steering, while minor discrepancies likely arise from fabrication tolerances and near-field coupling effects unaccounted for in simulations.

For the dual-beam splitter, normalized to a metal reference (*S*_11_ = − 13.8 dB), the two beams at ±39° achieve efficiencies of 44.67% and 35.48%, respectively, for a combined efficiency of 80.15%. This high total conversion confirms the effectiveness of the design.

#### SH-GSL engineered three-channel retroreflection

To evaluate the performance of the three-channel retroreflector, we conducted three experiments to measure the retroreflective angles, the perfect characteristics, and the efficiency of retroreflection.

In the first experiment, the far-field radiation performance of the retroreflector at different retroreflective angles is measured. As shown in Fig. 4c, a single horn, fixed to the rotating arm, serves as both the transmitting source and the receiver for reflected signals. The rotating horn illuminates the metasurface from various angles and scans across the retroreflective angles (Fig. 4m). The far-field radiation results demonstrate retroreflection at three distinct angles (Fig. 4p). The measured retroreflective angles (red rectangle) are approximately ±38° and 0°, which align with the theoretical (blue solid lines) and simulated (yellow dash lines) predictions. The discrepancies arise from the limitations in processing accuracy and experimental measurements.

The second experiment further verifies the perfect characteristics of the retroreflector. Figure 4a illustrates the experimental setup, where one horn is fixed to transmit at the retroreflective angle, and the other is rotated to receive the reflected signal at different positions. The metasurface is again placed vertically on the turntable. As seen in the inserts of Fig. 4q, s, apart from the mirror mode of −1st harmonic, only the corresponding harmonics are reflected into free space, with the mirror mode radiating much less energy. When retroreflected at the mirror mode, other spatial harmonics are suppressed, as shown in the insert of Fig. 4s. Figure 4q–s demonstrates that nearly no reflected signal is received at other positions, confirming the absence of undesired spatial harmonics in free space. It should be noted that certain angles cannot be measured due to the blind angle between the two arm supports, as shown in Fig. 4b. In these cases, the far-field pattern within the blind angle is replaced by the far-field radiation obtained from the single horn.

In the third experiment, a metal plate replaces the metasurface to assess the retroreflector’s efficiency. We quantify multi-channel retroreflection using a dual-horn antenna configuration (Fig. 4a), measuring transmission coefficients (*S*_21_) at three critical angles—Metasurface: −16.92 dB (−38°), −18.61 dB (+38°), −16.98 dB (0°); Metal plate: −16.88 dB (−38°), −17.81 dB (+38°), −15.5 dB (0°). The metasurface’s retroreflective efficiency closely matches the metal plate (≤2.1 dB variation), demonstrating high-efficiency multi-channel retroreflection. The metasurface achieves retroreflection efficiencies of 99.08% (−38°), 83.18% (+38°), and 71.12% (0°), demonstrating high-efficiency multi-channel retroreflection across multiple angular channels. Notably, the angular dependence of retroreflection efficiency arises from distinct harmonic coupling mechanisms: Peak efficiency at −38° corresponds to excitation of the fundamental harmonic (*ℓ* = 0), optimized by momentum conservation in the radiative regime; Reduced efficiency at +38° results from −2nd spatial harmonic dominance, as a higher-order mode naturally exhibiting lower radiative efficiency compared to the fundamental harmonic; Efficiency dip at 0° results from parasitic surface wave excitation (Fig. S8), where part of incident energy converts to plasmonic modes rather than retroreflection. The performance comparison of some existing retroreflectors is presented in Table 3, demonstrating the advantages of our design of the three-channel retroreflector, including extremely high retroreflective efficiency, a large retroreflective angle, and a structure that is ultrathin and single-layer.

**Table 3:** Retroreflective performance comparison

Ref.	Operating frequency	Polarization	Configuration	Efficiency	Maximum retro. angle
^32^	Visible-NIR	Polarization insensitive	Bulky	77.5% uncoated63.6% coated	near 0°
^37^	Near-IR (1550 nm)	Linear polarization	Cascaded	78% at normal incidence	25°efficiency 50%
^41^	8-10 GHz	Circular polarization	Single layer	80%	14.5°
This work	14 GHz	Linear polarization	Single layer	99.08%	39°

The bandwidth analysis of the dual-beam splitter and three-channel retroreflector can be found in Supplementary Material S3. This angular-specific performance, achieved through harmonic-selective phase engineering, validates the SH-GSL framework’s capability for simultaneous multi-channel retroreflection control—a critical advance for compact beam-routing systems.

## Discussion

The proposed spatial harmonic-expanded generalized Snell’s law (SH-GSL) fundamentally redefines metasurface engineering by unifying GSL with Floquet periodicity, thereby bridging the critical gap between harmonic dynamics and practical device design. Traditional GSL frameworks, constrained by their focus on the fundamental harmonic, inherently limit metasurfaces to single-channel functionalities. By contrast, SH-GSL introduces a deterministic framework that treats spatial harmonics as independent degrees of freedom, enabling simultaneous multi-channel wavefront manipulation. Unlike conventional metagratings that typically target a single diffraction order through sparse scatterer arrangements, SH-GSL analytically leverages periodic phase-gradient metasurfaces to fold the incident momentum into multiple discrete spatial harmonics, enabling systematic multi-channel functions (e.g., three-channel retroreflection, multi-beam splitting) with high efficiency. Compared to holographic metasurfaces relying on iterative optimization of phase profiles for farfield pattern reconstruction, SH-GSL provides direct analytic design rules based on compensation wavevector and Floquet expansion, simplifying design and offering clear criteria for harmonic excitation or suppression. Additionally, SH-GSL naturally unifies far-field control and near-field surface-wave excitation in one Floquet-harmonic perspective, a capability less explored in prior works. This paradigm shift is validated through three experimentally demonstrated applications—Floquet-engineered abnormal spatial-harmonic reflection, Floquet-engineered multi-beam splitting, and Floquet-engineered multi-channel retroreflection—which collectively showcase unprecedented control over harmonic-specific angular dispersion and efficiency.

While the three illustrative cases presented in this study empirically demonstrate the validity of the proposed SH-GSL, it is crucial to emphasize that the theory’s application scope extends substantially beyond these specific instances. The foundational principles and methodological constructs embedded within this theoretical model possess inherent generalizability, enabling their adaptation to diverse design scenarios across multiple domains.

A key distinction of SH-GSL lies in its decoupling of harmonic dynamics from meta-atom complexity. Unlike conventional approaches relying on multi-resonant meta-atoms or heuristic optimization, our framework establishes harmonic properties as a direct consequence of global periodicity and phase symmetry. This insight aligns with recent advances in Floquet-engineered metasurfaces but extends their scope by introducing a compensation wavevector principle that analytically links harmonic excitation to incident conditions. For instance, the three-channel retroreflector achieves up to 99% efficiency by selectively exciting desired harmonics while suppressing undesired modes to the near field, a feat unattainable through traditional grating-based designs. Such performance underscores the framework’s ability to harmonize momentum-space physics with real-world device constraints.

The experimental results reveal minor deviations between theoretical predictions and measured angles (e.g., ±38° vs. simulated ±39°), attributable to fabrication tolerances and near-field coupling effects. These discrepancies highlight the importance of refining meta-atom design to mitigate parasitic interactions, particularly for higher-order harmonics. Furthermore, while SH-GSL enables perfect retroreflection for three channels, extending this to higher channel counts remains challenging due to intrinsic momentum conservation constraints. This limitation suggests a trade-off between channel multiplicity and harmonic purity, necessitating future exploration of hybrid designs combining SH-GSL with dynamic tuning mechanisms. SH-GSL opens transformative avenues for applications demanding multi-channel parallelism. In next-generation communications, ultra-dense beamforming could exploit harmonics as orthogonal data carriers, vastly improving spectral efficiency. The SH-GSL framework—folding a linear phase gradient into discrete spatial harmonics via Floquet analysis—relies only on the metasurface providing prescribed phase shifts across a supercell. As a momentum-space formalism, it is frequency-agnostic in principle: one can apply the same design rules for Δ*k*_*∥*_ and supercell period at optical or other bands. However, practical implementation must consider material dispersion, losses, and nanofabrication constraints unique to each frequency range.

In conclusion, SH-GSL transcends the conventional trial-and-error paradigm, offering a physics-driven roadmap for metasurface innovation. By conceptualizing spatial harmonics as manipulable resources rather than parasitic interference, this work establishes a universal foundation for advanced wavefront engineering, poised for breakthroughs across ultra-dense beamforming, sensing, and meta-photonics through harmonic-division multiplexing.

## Methods

### Negative phase gradient

A negative phase gradient means the phase decreases along the +*x* direction (assuming the phase gradient along the +*x* direction), corresponding to a reversed direction of in-plane momentum compensation. In Floquet terms, this simply flips the sign of the momentum increment *G* = 2*π*/*P*, so that modes labeled by *ℓ* shift accordingly: e.g., what was *ℓ* + 1 = +1 for a positive gradient becomes *ℓ* + 1 = −1 for the reversed case, etc. Since the mirror mode is defined by *ℓ* + 1 = 0, the equivalent phase gradient of this mode is unsigned, so whether the phase gradient is positive or negative does not affect the order definition of this spatial harmonic.

### Numerical simulation

All numerical simulations are performed using the Frequency Domain Solver, which employs the Finite Element Method within COMSOL. To calculate the phase-amplitude properties of meta-atoms, the meta-atom is modeled with Floquet periodic boundary conditions in both the *x* and *y* directions. First, by sweeping the range of the frequency from 4 to 24 GHz, the properties of meta-atoms are obtained in the broad frequency range. Then, the designed frequency is selected, where the phase coverage is satisfied around 2*π*. Finally, four meta-atoms are selected to satisfy the phase gradient of *π*/2.

To calculate the electromagnetic response of three metasurfaces, each metasurface with an elaborate arrangement of the meta-atoms under periodic boundary conditions is simulated in both the *x* and *y* directions. According to the functionality of each metasurface (abnormal reflection, beam splitting, and retroreflection), the incident plane waves are illuminated on the metasurface. The distributions of the reflective electric fields are obtained by using the relative electric field. Moreover, the far-field patterns are calculated by setting the Far-Field Domain above the metasurface.

### Design rationale for selecting phase gradient

We note that each meta-atom in the supercell provides a distinct phase shift to the incident wave. By arranging these meta-atoms in a linear phase sequence (i.e., implementing a constant phase gradient), the metasurface array acquires a compensation wavevector determined by the phase distribution ΔΦ, the period *p*, and the operating frequency *f*_0_. Under the proposed SH‑GSL framework, for a given compensation wavevector and incidence angle, different spatial-harmonic orders exhibit distinct radiative or localized behavior, each with a uniquely determined far-field radiation angle. Therefore, the phase distribution of the meta-atoms in the gradient metasurface directly determines the compensation wave vector of the metasurface, thereby controlling the behavior of different spatial harmonics in the farfield (reflective angles) under different incident angles.

The selection of phase gradient (e.g., *π*/2) is not arbitrary but is rigorously tied to the desired functionality. As shown in Fig. 2b, different transverse compensation wave vectors Δ*k*_*∥*_ correspond to the selection of radiation spatial harmonics, where Δ*k*_*∥*_ is closely related to the phase gradient (Δ*k*_*∥*_ = ΔΦ/Δ*x* = Δ*φ*/*p*, Δ*φ* is the phase difference between adjacent meta-atoms, *p* is the period of the meta-atom).

In the case of the designed three-channel retroreflection, to reduce the effect of the undesired spatial harmonics, only three spatial harmonics (−2nd, −1st, and 0th) are utilized to radiate into free space for “perfect” three-channel retroreflection. Therefore, a transverse compensation wave vector Δ*k*_*∥*_ > *k*_0_ needs to be provided through the designed metasurface, and the phase difference *π*/2 between adjacent meta-atoms (the period of the meta-atom is *p* = 4.25 mm, Δ*k*_*∥*_ ≈ 1.26*k*_0_) exactly meets this demand. According to the proposed SH-GSL, the retroreflective angles corresponding to spatial harmonics can be calculated as (–39°, 0th), (+39°, −2nd), and (0°, −1st).

In the case of the designed beam splitter, after determining the designed angle of incidence (e.g., 0°), the transverse compensation wave vector can be designed according to the desired number of split beams. Under normal incidence, dual-beam splitting can be achieved utilizing two spatial harmonics (0th and −2nd); quad-beam splitting can be achieved utilizing four spatial harmonics (−3rd, −2nd, 0th, and 1st). With the designed period of the meta-atom *p* = 4.25 mm, the phase difference *π*/4 (Δ*k*_*∥*_ ≈ 0.63*k*_0_) and *π*/6 (Δ*k*_*∥*_ ≈ 0.42*k*_0_) exactly meet the demands of the dual-beam splitting and quad-beam splitting, respectively.

The proposed SH-GSL is a general design theory for gradient metasurfaces, with the selection of the phase gradient depending on the parameters of the meta-atoms (e.g., the period of the meta-atom) and the desired functionalities (e.g., the angles of the incidence and the reflection/transmission).

### Design rationale for choosing repeating-cell metasurface

The SH-GSL we proposed aims to fully utilize spatial harmonics. With consideration of multi-channel (i.e., multi-harmonic) functionalities, the excitation of high-order spatial harmonics is involved: the more channels are desired, the higher-order spatial harmonics that need to be excited. Therefore, we need the metasurface to be able to excite spatial harmonics as much as possible. The design principle of repeating-cell metasurface: the larger the phase gradient between adjacent meta-atoms, the stronger the nonlocal effect, and the more capable it is of stimulating spatial harmonics. By using two meta-atoms, the phase gradient between them is *π*, and the nonlocal effect is significantly stronger. The repeated arrangement is to construct different phase gradients: for example, the repeating-cell arrangement of 2 + 2 (two repeating ‘0’ meta-atoms and two repeating ‘*π*’ meta-atoms in one supercell) can be regarded as 2*π*/4, i.e., the phase difference of *π*/2; the repeating-cell arrangement of 4 + 4 (four repeating ‘0’ meta-atoms and four repeating ‘*π*’ meta-atoms in one supercell) can be regarded as 2*π*/8, i.e., the phase difference of *π*/4. In this case, each *N*_r_ repeated meta-atom forms a larger unit (the period of the larger unit is *P*_r_ = *N*_r_*p*) whose reflection/transmission phase is consistent with each meta-atom. In this way, we can construct metasurfaces with different phase gradients using only these two meta-atoms (Δ*k*_*∥*_ = Δ*φ*/*p* = *π*/*P*_r_ = *π*/*N*_r_*p*), and enhance nonlocal effects as well as the excitation of spatial harmonics.

### Experimental setup

Firstly, the metasurfaces mentioned above are fabricated using Printed Circuit Board (PCB) technology (56 × 56 meta-atoms for abnormal harmonics reflection, 56 × 56 meta-atoms for dual-beam splitter, 40 × 40 meta-atoms for three-channel retroreflector). Then, the metasurface sample is fixed vertically on the turntable, and the non-metasurface area is covered with absorbing materials. Note that all experiments are performed in the anechoic chamber.

In the first experiment, a single horn is fixed on the rotating arm as a wave source to illuminate the metasurface and receive the reflected signal at the same time. Subsequently, the data of *S*_11_ can be obtained by rotating the horn from −60° to 60°. Note that due to the existence of highly efficient retroreflection, a traveling standing wave is formed between the transmitting horn and the metasurface. Therefore, the transmitting horn needs to be placed at the peak of the standing wave to ensure the highest amplitude reception.

In the second experiment, two horns are fixed on the rotating arms to transmit the incident wave and receive the reflected wave, respectively. Fixing one horn at retroreflective angles (−39°, 0°, and 39°), the data of *S*_21_ can be obtained by rotating another horn from 90° to −90°. Note that the reflection conditions at some angles cannot be measured due to the blind angle between the two arm supports. Here, the far-field pattern in the range of blind angle is replaced by the far-field radiation obtained by the single horn.

In the third experiment, the metasurface is replaced with a metal plate, and the non-metasurface area is still covered with absorbing materials, ensuring the area of absorbing materials is constant. Fixing one horn at retroreflective angles (−39° and 39°), the data of *S*_21_ can be obtained by fixing another horn at mirror positions (39° and −39°), and the data of *S*_11_ is obtained at 0° by one horn. Considering these data as the total energy of the transmitted signal, the efficiency of retroreflection can be calculated.

## Supplementary information

Supplementary Information

## Data Availability

All data presented within the article and Supplementary Materials are available from the corresponding author upon reasonable request.
