# Supplementary material for: Missing harmonic dynamics in generalized Snell’s law: revealing full-channel characteristics of gradient metasurfaces
Source: Light Sci Appl. 2025 Sep 15;14:321. doi: 10.1038/s41377-025-02009-3 (PMC12434143; doi:10.1038/s41377-025-02009-3)
Supplement: Supplementary file 1 — Supplementary Information [file 41377_2025_2009_MOESM1_ESM.docx]

Title page

Supplementary Information for Missing Harmonic Dynamics in Generalized Snell’s Law: Revealing Full-Channel Characteristics of Gradient Metasurfaces

Yueyi Zhang^1^, Fengyuan Han^1^, Yibing Xiao^1^, Ziwen Zhang^1^, Jitao Yang^1^, Yulu Lei^1^, Fei Gao^2^, **Hongsheng Chen^2, *^**, & **Chao-Hai Du^1, *^**

^1^State Key Laboratory of Photonics and Communications, School of Electronics, Peking University, Beijing 100871, China

^2^Key Lab. of Advanced Micro/Nano Electronic Devices & Smart Systems of Zhejiang, College of Information Science and Electronic Engineering, Zhejiang University, Hangzhou 310058, China

^*^Correspondence author: [**hansomchen@zju.edu.cn**](mailto:hansomchen@zju.edu.cn)**;** [**duchaohai@pku.edu.cn**](mailto:duchaohai@pku.edu.cn)

S1: Energy distribution of spatial harmonics

- **Mode Strength Prediction via Floquet-Based Fourier Analysis**

Without considering nonlocal effects between meta-atoms (Local Periodic Approximation), the energy distribution among spatial harmonics is analyzed using the Floquet-Bloch theory, where the reflected field is decomposed into a Fourier series of spatial harmonics. For a gradient phase metasurface with periodically arranged meta-atoms along the *x*-direction, the reflected electric field *E*(*x*) under incidence at *θ*_i_ can be expressed as:

$$E(x)=\sum_{m=-\infty}^{+\infty} A_{m}(x)\cdot\exp\left\{-j\left[\Phi(x)+k_{0x}x+\frac{2\pi m}{Np}x\right]\right\}$$

where *m* represents the order of the spatial harmonic, *A_m_*(*x*) is the complex amplitude of the *m*-th spatial harmonic, Φ(*x*) represents the phase distribution along the *x* direction, and *k*_0_*_x_* = *k*_0_sin*θ*_i_ is the projection of the incident wave vector in the *x* direction. Utilizing the principle of orthogonality, *A_m_*(*x*) can be derived as follows:

$$A_{m}=\frac{1}{Np}\int_{-Np/2}^{Np/2} E(x)\cdot\exp\left\{j\left[\Phi(x)+k_{0x}x+\frac{2\pi m}{Np}x\right]\right\}dx$$

Generally, gradient metasurfaces have a constant phase difference Δ*φ* between adjacent meta-atoms, and the phase wrapping results in a phase change of 2*π* within one period, thus Φ(*x*) = (Δ*φ*/*p*)*x* = (*N*Δ*φ*/*Np*)*x* = (2*π*/*Np*)*x*. Assuming an ideal situation with no losses and the total energy of 1 (i.e., *E*(*x*) = 1), the complex amplitude of *m*-th spatial harmonic can be expressed as:

$$A_{m}=\frac{1}{Np}\int_{-Np/2}^{Np/2} \exp\left\{j\left[k_{0}\sin\theta_{\mathrm{i}}+\frac{2\pi\left(m+1\right)}{Np}\right]x\right\}dx=\frac{\sin\left(k_{\mathrm{total}}Np/2\right)}{k_{\mathrm{total}}Np/2}$$

where *k*_total_ = *k*_0_sin*θ*_i_ + 2*π*(*m*+1)/*Np*. According to this formula, the energy distribution of *m*-th spatial harmonic can be obtained by *η_m_* = *A_m_*^2^. Therefore, the energy distribution of different spatial harmonics is related to the incident angle *θ*_i_ and the phase gradient Δ*φ* of the metasurface.

In the case of the designed three-channel retroreflection with Δ*φ* = *π*/2 and *N* = 4, the energy distribution of the spatial harmonics mainly utilized (−2nd, −1st, and 0th) is illustrated in **Fig. S1**. The energy distribution of the mirror mode is dominant only around 0° incidence. As the incident angle increases, its energy proportion will gradually decrease, while the spatial harmonics corresponding to the reflection channel will obtain more energy distribution at the retroreflective angle.

This analysis assumes local periodicity, where the nonlocal effect between meta-atoms is negligible. However, when adjacent meta-atoms exhibit a phase difference Δ*φ* > *π*/4, nonlocal effects (e.g., near-field coupling) become significant, invalidating the local periodic approximation. For metasurfaces composed of complex meta-atoms, rigorously modeling these effects requires the combination with the aforementioned Floquet theory and full-wave simulations or advanced coupling-matrix theories—coupled mode theory (CMT) or spatio-temporal coupled mode theory (SCMT), which are beyond the scope of this analytical framework.

- **Simulation Validation of Energy Distribution**

We have performed additional full-wave simulations by setting diffraction orders in COMSOL to extract the reflectivity in each radiative order for representative designs, utilizing periodic boundary conditions and periodic ports.

The finite element method in COMSOL and Rigorous Coupled-Wave Analysis was employed to calculate the spatial harmonic properties of the designed meta-atoms and metasurfaces, including the abnormal single-side reflection, three-channel retroreflection, five-channel retroreflection, dual-beam splitting, and quad-beam splitting. For period structures (supercells), the periodic boundary conditions were applied along the *x*- and *y*-direction. The periodic ports were applied to the two ends in the *z*-direction, with setting diffraction orders to calculate the coupling of spatial harmonics.

In the case of the three-channel retroreflection, the gradient metasurface with a phase gradient of *π*/2 was adopted, where the diffraction orders (0th, −1st, and −2nd) were applied. The reflectivities of each spatial harmonics are shown in **Fig. S2**a. It can be demonstrated that at the angle of retroreflection (the retroreflective channel), the corresponding spatial harmonic is dominant (0th spatial harmonic at −39° with blue line, −1st spatial harmonic at 0° with yellow line, and −2nd spatial harmonic at 39° with red line). Additionally, within the range of −15.1° to +15.1°, the mirror mode (−1st spatial harmonic) is dominant, while near the retroreflection angle, the mirror mode is suppressed to a smaller magnitude, and thus can be neglected when discussing the retroreflection of non-mirror modes. Notably, among the three retroreflection modes, the mirror mode has the highest retroreflection energy, followed by the 0th spatial harmonic, and finally, the −2nd spatial harmonic, resulting in an approximately symmetrical spatial harmonics distribution.

In the case of the abnormal single-side reflection, the repeating-cell metasurface with 2 + 2 meta-atoms (*N* = 4, *N* is the number of meta-atoms in one period) was adopted, where the diffraction orders (0th, −1st, and −2nd) were applied. The reflectivities of each spatial harmonics are shown in **Fig. S2**b. It can be demonstrated that as the incident angle changes, the energy distribution among the three spatial harmonics (0th, −1st, and −2nd) exhibits an alternating dominant trend, consistent with the simulation results we demonstrated in the supplementary materials. Notably, this repeating-cell metasurface can also be utilized to achieve the same three-channel retroreflection as in **Fig. S2**a, but with a symmetric spatial harmonics distribution.

In the case of the five-channel retroreflection, the gradient metasurface (**Fig. S2**c) with the phase gradient of *π*/3 and the repeating-cell metasurface (**Fig. S2**d) with 3 + 3 meta-atoms (*N* = 6) were both simulated utilizing COMSOL. Each retroreflective angle needs to utilize different spatial harmonics to achieve retroreflection, meaning that each spatial harmonic corresponds to only one retroreflective channel. According to the proposed SH-GSL, the retroreflective channels of the five-channel retroreflection are (0°, −1st), (−24.8°, 0th), (24.8°, −2nd), (−57.2°,1st), and (57.2°, −3rd), which are all marked with red vertical regions. It can be demonstrated that the energy distribution of spatial harmonics in the gradient metasurface is asymmetric with some retroreflective channels closed (−57.2°,1st) and (0°, −1st), while the energy distribution of spatial harmonics in the repeating-cell metasurface is symmetric with all retroreflective channels open. Although the five-channel retroreflection is not perfect and certain spatial harmonics may not dominate in the corresponding retroreflective channels, the results still demonstrate the ability of the repeating-cell metasurfaces to enhance spatial harmonics.

In the case of the dual-beam splitting and the quad-beam splitting, the repeating-cell metasurfaces (4 + 4 meta-atoms with Δ*k* = 0.63*k*_0_ for dual-beam splitting in **Fig. S2**f, 6 + 6 meta-atoms with Δ*k* = 0.42*k*_0_ for quad-beam splitting in **Fig. S2**h) were adopted. It can be demonstrated that the 0th and −2nd spatial harmonics are used to form symmetric dual-beam splitting with minimal mirror reflection in the design dual-beam splitting; the 0th and −2nd, 1st and −3rd spatial harmonics are used to form symmetric quad-beam splitting, with the 0th and −2nd spatial harmonics having the highest energy, the 1st and −3rd spatial harmonics having lower energy, and the mirror reflection having the lowest energy.

The results confirm that the retroreflection channel indeed carries the majority of the power, whereas the mirror mode is suppressed around the retroreflective channels. Besides, the energy distributions of spatial harmonics in all the designed metasurfaces are calculated and simulated to predict the relative strength of the radiation into specific radiative channels.

S2: Analysis of the mirror mode

As mentioned above, the mirror reflection mode associated with l = −1 always exists. Once the metasurface design is completed, it is essential to evaluate the proportion of the mirror mode in each retroreflective channel, i.e., to determine whether the mirror mode is the prevailing one. This implies that the compensating wave vector Δ*k_∥_* of the metasurface is already known and fixed, and it is necessary to calculate a specific range of incident angles within which the mirror mode prevails. Furthermore, the influence of mirror mode within retroreflective channels beyond the specified range can be effectively considered negligible. In the following, the specific range of incident angles is discussed.

The SH-GSL can be written further as

Based on the above, it can be concluded that the three-channel retroreflector is intrinsically the perfect retroreflector. Therefore, only the case of the perfect three-channel retroreflection is discussed here, i.e., l + 1 = 0, ±1. To calculate the domination of the mirror reflection mode, other orders of spatial harmonics should not exist in free space. Thereby, it can be derived using the expression

Then, it can be written further as follows:

By solving these two inequations, it can be deduced as

According to the range Δ*k_∥_* of the perfect multi-channel retroreflector in **Results**, the range of incident angles within which the mirror reflection mode is dominant in three-channel retroreflectors can be derived using the expression

$$\left|\sin\theta_{\mathrm{i}}\right|\in\left[0,-1+\left|\frac{\Delta k_{//}}{k_{0}}\right|\right]$$

(i). In the case of positive Δ*k_∥_* (Δ*k_∥_* ≥ 0), according to and , it can be derived as follows:

Thus, since the range of the compensation wave vector of the three-channel retroreflector is Δ*k_∥_* ∈ [*k*_0_, 2*k*_0_], the equation above can be written further using the expression

$$\left|\sin\theta_{\mathrm{i}}\right|\in\left[0,-1+\frac{\Delta k_{//}}{k_{0}}\right], \Delta k_{//}>0$$

(ii). Similarly, it can be derived in the case of Δ*k_∥_* < 0 as

$$\left|\sin\theta_{\mathrm{i}}\right|\in\left[0,-1-\frac{\Delta k_{//}}{k_{0}}\right], \Delta k_{//}<0$$

In conclusion, the range of incident angles within which the mirror reflection mode is dominant in three-channel retroreflectors can be derived using the expression

$$\left|\sin\theta_{\mathrm{i}}\right|\in\left[0,-1+\left|\frac{\Delta k_{//}}{k_{0}}\right|\right]$$

Note that in the case of the perfect retroreflection proposed in this paper, all other spatial harmonics do not exist in the radiation space, except for the mirror mode and retroreflective mode. Furthermore, the mirror mode dominates the main reflective mode, within the area of incident angles as described by . Beyond this area, although the mirror mode still exists, it is much lower compared to the retroreflective mode, that is, the effect of the mirror mode can be negligible.

S3: Broadband analysis of different functionalities

Indeed, while our meta-atom exhibits a broadband phase response from 10 to 23 GHz, the multi-channel functionalities (e.g., three-channel retroreflection, beam splitting) rely not only on the availability of phase coverage but also on the precise compensation wavevector Δ*k_∥_* and the relative phase accuracy across the operating band. Below, we summarize additional simulation-based bandwidth analyses and, where available, measurement insights.

1. **Distinction Between Meta-Atom Phase Bandwidth and Functional Bandwidth**

- The phase response of the meta-atom (10–23 GHz) indicates that for a single meta-atom, the required discrete phase states (e.g., 0, *π*/2, *π*, 3*π*/2) can be realized approximately over a wide band. However, multi-harmonic functionalities depend on coherent interference among many meta-atoms arranged in a supercell, and on the condition that the compensation wavevector Δ*k_∥_* = Δ*φ*/*p* produces the intended set of radiative harmonics.
- We note that when the operating frequency deviates from the design frequency (14 GHz), the individual phase responses of the meta-atoms shift so that the intended linear phase arrangement across each supercell is no longer exact (**Fig. S10**). Since the gradient-metasurface functionality relies critically on this phase distribution (i.e., precise Δ*k_∥_*), any phase deviation alters the effective compensation wavevector and may move target spatial harmonics out of (or undesired harmonics into) the radiation cone. Consequently, efficiency degrades and, beyond a certain detuning, the designed function may not be realized at all. Moreover, the radiation or localized status of spatial harmonics may change with frequency, further affecting performance. We therefore perform frequency-sweep simulations to quantify how phase shifts reduce efficiency and narrow the operational bandwidth of each function.

1. **Additional Full-Wave Simulations for Bandwidth Assessment**

We performed full-wave COMSOL simulations for each primary function (three-channel retroreflector, dual- and quad-beam splitters) over a frequency sweep around 14 GHz. Specifically, we simulated from 12 GHz to 18 GHz in steps of 1 GHz under periodic boundary conditions to demonstrate each functionality at each frequency.

- Dual-beam splitting

We have carried out both theoretical angle calculations and full-wave COMSOL simulations over 12-18 GHz using the original phase-gradient design (Δ*φ* = *π* between adjacent meta-atoms).

Keeping the phase gradient fixed at *π*, the transverse compensation wavevector Δ*k_∥_* varies with frequency, yielding a frequency-dependent splitting angle as predicted by SH-GSL. **Fig. S11a** shows the calculated beam-splitting angle across 10-20 GHz, with different frequencies exhibiting distinct splitting angles.

At the designed frequency of 14 GHz, the simulated far-field pattern matches the theoretical splitting angle within 2° error approximately (**Fig. S11b**). Over 12-18 GHz, the metasurface still produces beam splitting, and the observed splitting angles generally follow the theoretical curve from **Fig. S11a**. However, performance varies with frequency, e.g., splitting occurs at 12 GHz but with reduced efficiency and larger angular deviation (**Fig. S11c**) compared to 14 GHz; The efficiency drops sufficiently that clear dual-beam splitting is not realized at 15 GHz (**Fig. S11e**).

These results demonstrate that, although individual meta-atoms exhibit a broadband phase response, the supercell phase arrangement is optimized at 14 GHz; detuning leads to phase deviations that alter Δ*k_∥_* and the radiation condition for the chosen harmonics, causing efficiency degradation or functional failure at certain frequencies.

Without explicit broadband meta-atom optimization, the designed dual-beam splitter maintains functionality over a moderately wide band (approximately 12-18 GHz) but with decreasing efficiency and increasing angle error away from 14 GHz and may fail to split cleanly at some frequencies (e.g., around 15 GHz). This behavior is consistent with the dependency of the exact phase distribution on frequency.

- Three-channel retroreflection

With the same approach used for the dual-beam splitter, we kept the phase gradient Δ*φ* = *π* fixed and carried out. Over 10-20 GHz, we computed the expected retroreflection angles under SH-GSL. Due to symmetry, we focus on one side (39° at 14 GHz). The theoretical curve shows how this angle shifts slightly with frequency as Δ*k_∥_* changes (**Fig. S12a**).

At the designed frequency of 14 GHz, the simulated far-field pattern exhibits a pure retroreflection at ~39° with ~1.4° error versus theory, high efficiency, and a clean radiation lobe (**Fig. S12b**).

Retroreflection is still observed near the theoretical angle at 12 GHz but with noticeable mirror reflection, reducing net retroreflector efficiency (**Fig. S12c**); Mirror reflection increases further at 15 GHz, leading to a lower retroreflection amplitude (**Fig. S12e**); Mirror reflection becomes larger than the retro-reflection (**Fig. S12g and h**), so the retroreflective efficiency is greatly reduced or the retroreflection may fail; At intermediate frequencies (13-16 GHz), retroreflection persists but with gradually reduced purity and efficiency as frequency moves away from 14 GHz.

Although the meta-atoms exhibit a broad phase range, the three-channel retroreflector’s functionality relies on precise phase arrangement to place only the intended harmonic in the radiation cone. Frequency detuning alters individual element phases, causing increased undesired mirror reflection and thus degraded efficiency/purity at other frequencies. Nevertheless, a clear retroreflection lobe appears over a moderately wide band around 14 GHz. This confirms that while not explicitly broadband-optimized, the three-channel retroreflector maintains its function over a significant range with trade-offs in efficiency and lobe purity.

1. **Discussion of Bandwidth-Limiting Factors**

- **Phase dispersion of meta-atom:** Although the metasurface elements exhibit phase responses across the entire broadband, the actual phase state deviates from the designed value when the frequency deviates from 14 GHz; this phase error alters the metasurface's compensation wave vector Δ*k_∥_*, thereby changing the actual realized functionality.
- **Radiation condition shift:** Since *k*_0_ = 2*πf*/*c* changes with frequency, the set of radiative harmonics for a given Δ*k_∥_* can change: some harmonics may exit or enter the radiation cone, altering the multi-channel behavior.
- **Inter-element coupling variation:** Frequency-dependent coupling modifies the amplitude and phase response of each meta-atom in the supercell, affecting the interference pattern.

S3: Abnormal spatial-harmonic reflection

The structure of the metasurface and the simulation results are shown in **Fig. S3**.

S4: Multi-beam splitting

The structure of the quad-beam splitter and the simulation results are as illustrated as **Fig. S4**.

S5: Analysis of the multi-channel retroreflection and the perfect multi-channel retroreflection

Based on the above analysis of the extension, the design method of multi-channel retroreflectors utilizing single-layer periodic metasurfaces is discussed here.

As illustrated in **Fig. 1d** left, both the positive and negative zones of the incidence and reflection are defined. As for a typical retroreflector, incident waves with both positive and negative incident angles can be retroreflected. Thus, the range of *θ*_i_ is selected from −*π* / 2 to *π* / 2. Then, with the condition of the retroreflection *θ*_i_ = −*θ*_r_, the order of retroreflected harmonics can be calculated as

As described in , the equation is invalid unless 2*k*_0_sin*θ*_i_ is integer multiples of Δ*k_∥_*, which guarantees that l is an integer. Depending on different integers, the values of *θ*_i_ can be different, which enables multi-channel retroreflection. Therefore, the incident angle *θ*_i_ can be written as the function of the l, i.e., *θ*_i_(l), which can be expressed as follows:

Assume that the compensation wave vector of the metasurface is designed as a specific value of Δ*k_∥_*, which guarantees that possesses one or more solutions. In this way, each solution corresponds to the specific retroreflective channel, as illustrated in **Fig. 1d** right.

(i) In the case of the single-channel metasurface retroreflector, only one solution of is valid. Then, the value of the incident angle can be derived

where

$$\left|\Delta k_{//}\right|\in\left[2k_{0},+\infty\right)$$

This means retroreflection only occurs at normal incidence in the case of l = −1. Note that the −1st spatial harmonic (l = −1) corresponds to the mirror reflection mode and always exists whatever the designed value of Δ*k_∥_* is. The range of Δ*k_∥_* will be derived in the following.

(ii) When three solutions of exist, three retroreflective channels will appear. Except for the retroreflective channel solved in the case of (i), extra channels can be obtained by combining and l+1 = ±1. Thereby, the values of the incident angles for each channel can be deduced as follows:

Considering the range of sin*θ*_i_ with (−1, 1) and the case of five-channel retroreflection, the range of Δ*k_∥_* can be expressed as follows (the former corresponds to the upper bound, and the latter corresponds to the lower bound):

$$\left|\Delta k_{//}\right|\in\left[k_{0},2k_{0}\right)$$

where the upper bound in indicates the range of Δ*k_∥_* in . The lower bound in will be discussed in the following.

(iii) Concerning the five-channel retroreflector, the incident angles can be solved similarly:

where

$$\left|\Delta k_{//}\right|\in\left[\frac{2}{3}k_{0},k_{0}\right)$$

The upper bound in reveals the range of the compensation wave vector of the three-channel retroreflector in . Besides, the lower bound in will be calculated in the following.

(iv) Finally, the range of the incident angle for an *m*-channel (*m* = 2*n* − 1, *n* Z_+_) retroreflector can be further deduced following the aforementioned steps.

where

$$\left|\Delta k_{//}\right|\in\left[\frac{4k_{0}}{m+1},\frac{4k_{0}}{m-1}\right)$$

Note that the lower bound in can be used to calculate the range of the five-channel retroreflector (*m* = 5). The lower bound can be obtained according to the expression of the incident angle of the extra channel in the (*m* + 2)-channel retroreflector compared with the case of *m* channels.

In this way, the compensation wave vector of the m-channel retroreflector can be selected and designed according to . Simultaneously, the incident (retroreflective) angles, the corresponding retroreflective channels, and the harmonics can be calculated using .

In summary, the method to design an *m*-channel retroreflector is proposed, with the corresponding incident angle and the compensation wave vector provided by and . Based on the theory proposed in this section, a multi-channel retroreflector can be achieved by elaborately designing the Δ*k_∥_* and retroreflective angles can be also obtained as shown in **Fig. 1d** right. Furthermore, it can be concluded that the broad or continuous angle of the single-layer retroreflector can be achieved with more channels.

Here, the design purpose of the perfect retroreflector is presented: for each channel of the multi-channel retroreflector, only the spatial harmonic, which the specific retroreflective mode corresponds to, exists in this channel to generate pure retroreflections. This means that the other undesired spatial harmonics do not exist in the radiation field or are sufficiently suppressed around the reactive near field as shown in **Fig. 1d**. Note that the mirror reflection mode (l = −1) is inevitable during the whole retroreflection process. Therefore, the mirror reflection mode will be analyzed in the following part.

Assume that retroreflection occurs in the case of the incident angle of *θ*_i_, the order of the corresponding spatial harmonic is l_retro_, and the rest orders are l_other_. In this case, the retroreflective angle can be denoted as *θ*_i_(l_retro_) according to . Then, the perfect retroreflection can be achieved by suppressing undesired spatial harmonics as surface plasmons around the reactive near field. This means that l ∈ {l ≠ l_retro_} { l ≠ −1}, the following expression holds:

According to , the range of Δ*k_∥_* can be obtained to design the perfect retroreflector.

To better describe the case of the perfect multi-channel retroreflection, the method of Logical Multiplication is adopted here. Assuming that event *A* occurs under condition *B*, it can be expressed as *P*(*A* | *B*) = 1, otherwise *P*(*A* | *B*) = 0. Therefore, when event *A* occurs under all conditions *B*, *C*, and *D*, it can be expressed as

$$P(A|B)\cdot P(A|C)\cdot P(A|D)=1$$

where once the event *A* is invalid under any condition, the value of is equal to 0.

Accordingly, the expression in can be defined as event *A*, and condition *B* is l ∈ {l ≠ l_retro_} {l ≠ −1}, i.e.,

where l ∈ Z, and the condition {l ≠ −1} is due to the mirror-reflection mode that always exists. Furthermore, *P*(*A* | *B*) expressed by could represent whether the case of the retroreflection corresponding to l_retro_ is perfect or not. Note that, *P*(*A* | *B*) = 1 indicates that for any arbitrary l except for l_retro_, the inequality in the event *A* holds, i.e., the case of the retroreflection corresponding to l_retro_ is perfect; 0 < *P*(*A* | *B*) < 1 represents that there exists at least one spatial harmonic l in the radiation field resulting in the imperfect retroreflection; *P*(*A* | *B*) = 0 signifies that all spatial harmonics l are radiated into free space, which leads to the imperfect retroreflection.

In addition, since each incident angle (retroreflective angle) *θ*_i_ corresponds to the specific l-th spatial harmonic, the order of spatial harmonic can be expressed as a function of the incident angle, i.e., l(*θ*_i_). As for the perfect retroreflector, at the incident angle *θ*_i_, only l(*θ*_i_)-th harmonic can be retroreflected, and others are suppressed as surface plasmons. Therefore, the range of the compensation wave vector of the perfect all-channel (*m*-channel) retroreflector needs to be satisfied according to as:

where can be calculated according to . Next, according to , the range of Δ*k_∥_* of the single-, three-, and five-channel retroreflector will be discussed, and further extended to the *m*-channel retroreflector.

(i). *Discussion of the single-channel retroreflector*.

In the case of a perfect single-channel retroreflector, its retroreflection channel corresponds to the −1st spatial harmonic, in which the retroreflective angle can be deduced as *θ*_retro_ = 0°. According to SH-GSL, regardless of the transverse compensation wave vector provided by the metasurface, the retroreflection of the −1st spatial harmonic always exists. This means that the single-channel retroreflector corresponding to −1st spatial harmonic is always perfect, i.e., *P*[*A*(0)|*B*(0)] ≡ 1. Therefore, a perfect single-channel retroreflector must be designed based on the −1st spatial harmonic, while the radiations of all the other harmonics are suppressed around the reactive near field.

(ii). *Discussion of the three-channel retroreflector.*

As for a three-channel retroreflector, the perfect retroreflection is obtained by suppressing undesired harmonics into surface plasmons. Thus, the compensation wave vector Δ*k_∥_* of the designed metasurface can be derived by substituting to the item of as follows

To ensure that and always hold for l + 1 ≠ ±1 and l + 1 ≠ 0 in all situations, the compensation wave vector Δ*k_∥_* must satisfy the following expressions

Thus, in the case of the perfect three-channel retroreflector, the compensation wave vector of the metasurface must be satisfied

$$\left|\Delta k_{//}\right|\geq\frac{2}{3}k_{0}$$

Therefore, the range of the compensation wave vector can be finally obtained according to and as follows

$$\left|\Delta k_{//}\right|\in\left[k_{0},2k_{0}\right)$$

Then, it can be concluded that the three-channel retroreflector intrinsically functions as a perfect retroreflector.

(iii). *Discussion of the five-channel retroreflector*.

Similarly, the compensation wave vector of the perfect five-channel retroreflector needs to be satisfied as follows:

According to and , l = 0 and l = −2 are the maximum-value points of the right-hand side, respectively. However, the maximum value of the expression on the right-hand side will tend towards infinity, leading to the inequality invalid. Substantially, according to the corresponding expression of *θ*_i_(l = 1) and *θ*_i_(l = −3) in , the reflective angle *θ*_r_ could be calculated as 0° in both incidence cases, indicating that the vertical anomalous reflection always exists. Therefore, the five-channel retroreflector cannot be perfect. The design and simulation of the five-channel retroreflector can be seen in supplementary materials.

According to , the range of Δ*k_∥_* of the *m*-channel retroreflector is discussed in the following.

Furthermore, similar to the previous discussions, the range of the compensation wave vector of the metasurface for the *m*-channel retroreflector needs to be satisfied as follows:

Given that *m* denotes an odd number, it can be represented as

$$m=\left\{\begin{matrix} 4q+1 \\ 4q+3 \end{matrix}\right., q\in N$$

where N denotes the set of natural numbers. In the following, the range of Δ*k_∥_* will be discussed in two cases according to .

(i) In the case of *m* = 4*q* + 1, can be further written as

Hence, l = *q* − 1 is the maximum-value point of the right-hand side of the inequation above. Furthermore, if *q* ≠ 0, then the maximum value of the expression on the right-hand side will tend towards infinity, leading to the inequality invalid. As for the situation of *q* = 0, l = *q* − 1 = −1 is not located in the range of the parameter l in . Thereby, the expression on the right-hand side possesses the maximum value *k*_0_ for l = 0 or l = −2. Then, the range of Δk*_∥_* can be derived as follows

$$\left|\Delta k_{//}\right|\geq k_{0}, q=0, m=4q+1=1$$

In this case, the range of Δk*_∥_* is obtained at *m* = 1, which indicates the single-channel retroreflector. Considering the discussion of the single-channel retroreflector in , the final range of Δk*_∥_* can be derived as

$$\left|\Delta k_{//}\right|\geq2k_{0}, q=0, m=4q+1=1$$

(ii) In the case of *m* = 4*q*+3, it can be derived further based on

According to , the maximum value of the expression on the right-hand side is achieved for l = *q* or l = *q* − 1. Furthermore, if *q* ≠ 0, the maximum value of the expression on the right-hand side will be obtained at *q* or *q* − 1. Then, the range of Δ*k_∥_* can be derived as follows:

$$\left|\Delta k_{//}\right|\geq2k_{0}, q\neq0, m=4q+3\geq7$$

However, since the Δ*k_∥_* of that *m*-channel retroreflector will never exceed 2*k*_0_ according to , the situation described by fails to meet the design requirement. Moreover, the maximum values of the expression on the right-hand side will be obtained at *q* + 1 or *q* − 2 for *q* = 0 due to l ≠ 0 and l ≠ −1. Then, the range of Δ*k_∥_* can be derived as follows:

$$\left|\Delta k_{//}\right|\geq\frac{2}{3}k_{0}, q=0, m=4q+3=3$$

In this case, the range of Δk*_∥_* is obtained at *m* = 3, which indicates the three-channel retroreflector. Considering the discussion of the single-channel retroreflector in , the final range of Δk*_∥_* can be derived as

$$k_{0}\leq\left|\Delta k_{//}\right|<2k_{0}, q=0, m=4q+3=3$$

(iii) When *m* = 4*q* + 1, can be further written as

Hence, l = −*q* − 1 is the maximum-value point of the right side of the inequation above. Furthermore, if *q* ≠ 0, then the maximum value of the expression on the right-hand side will tend towards infinity, leading to the inequality invalid. As for *q* = 0, the point of l = −*q* − 1 = −1 is not located in the range of the parameter l in . Thereby, the expression on the right-hand side possesses the maximum value *k*_0_ at l = 0 or l = −2. Then, it can be derived as

$$\left|\Delta k_{//}\right|\geq k_{0}, q=0, m=4q+1=1$$

(iv). In the case of *m* = 4*q* + 3, it can be derived further based on

Thus, for , the maximum value of the expression on the right-hand side is achieved when it approaches the point nearest to l = −*q* − 3/2, denoted as l = −*q* −1 or l = −*q* − 2. Furthermore, if *q* ≠ 0, then the maximum value of the expression on the right-hand side will be attained at −*q* −1 or −*q* − 2. Then, the range of Δ*k_∥_* can be deduced as follows:

$$\left|\Delta k_{//}\right|\geq2k_{0}, q\neq0, m=4q+3\geq7$$

However, since the Δ*k_∥_* of that *m*-channel retroreflector will never exceed 2*k*_0_ according to , the situation described by fails to meet the design requirement. While the maximum value of the expression on the right-hand side will be attained at −*q* − 3 or −*q* with *q* = 0. Then, the range of Δ*k_∥_* can be derived as follows:

$$\left|\Delta k_{//}\right|\geq\frac{2}{3}k_{0}, q=0, m=4q+3=3$$

Finally, the range of Δ*k_∥_* determined by also can be derived as follows:

The details of the rest discussions can be seen in supplementary materials.

In summary, according to , , , , and , the compensation wave vector of the perfect *m*-channel retroreflector must be satisfied as:

$$\left|\Delta k_{//}\right|\in\left[k_{0},\infty\right)$$

Therefore, only single and three-channel retroreflectors can achieve perfect retroreflection, whereas a retroreflector featuring more than three channels cannot achieve perfect retroreflection.

S6: Design of the five-channel retroreflector

According to the design bias of the multi-channel retroreflector, the five-channel retroreflector is designed and simulated based on the meta-atoms proposed in this paper. The five-channel retroreflector is achieved using the five spatial harmonics (l + 1 = 0, ±1, ±2), and the compensation wave vector is designed by the limitation |Δ*k_∥_*|∈[2/3*k*_0_, *k*_0_). Then, six meta-atoms including three ‘0’ and three ‘*π*’ meta-atoms are arranged to construct the five-channel retroreflector, which results in a compensation wave vector of approximately 0.84*k*_0_, as depicted in **Fig. S5a**. According to , retroreflective angles of this retroreflector can be calculated as −24.8° (l = 0), 0° (l = −1), −57.2° (l = +1), 24.8° (l = −2), and 57.2° (l = −3).

The simulations with the five incidences are performed in COMSOL with periodic boundaries both in the *x* and *y* directions, and the results are shown in **Fig. S5**. From the simulation results, though five retroreflective channels are achieved, there are still other spatial harmonics existing in radiation space. There, these results demonstrate that the five-channel is an imperfect retroreflector.

S7: The method for calculating the far-field radiation

In this paper, a single horn is used to both transmit and receive electromagnetic waves, providing far-field radiation, which contrasts with the conventional approach that uses two horns to calculate the far-field pattern. To theoretically analyze the far-field radiation of the retroreflector, the method for calculating the far-field radiation is outlined here.

When the incident wave illuminates the metasurface, the reflected wave will be reflected into free space. If an infinite receiving plane is assumed, the received signal is the projection of the reflected wave onto this plane. As shown in **Fig. S6a**, when using a single horn for both transmission and reception, the ideal and lossless receiving signal, denoted as $S_{\mathrm{r}}^{\mathrm{ideal}}$, can be expressed as follows:

$$S_{\mathrm{r}}^{\mathrm{ideal}}=E_{\mathrm{t}}/E_{\mathrm{r}}=\cos\left(\left|\theta_{\mathrm{i}}+\theta_{\mathrm{r}}\right|\right)$$

where *E*_t_ and *E*_r_ represent the amplitudes of transmitted and reflected waves, respectively. It should be noted that the receiving signal is zero when the reflected wave is far from the receiving plane. Additionally, Eq. assumes an ideal and infinite receiving plane. In practice, the receiving plane is finite, determined by the aperture of the receiving antenna. Assuming that the directivity coefficient of the receiving antenna is *D*(*θ*), as described as follows:

$$D\left(\theta\right)=\frac{1+\cos\theta}{2}\cdot\frac{\sin\left(k_{0}a/2\sin\theta\right)}{k_{0}a/2\sin\theta}$$

where *a* is the aperture of the receiving antenna. Therefore, the real receiving signal, denoted as $S_{\mathrm{r}}^{\mathrm{real}}$, can be expressed as:

$$S_{\mathrm{r}}^{\mathrm{real}}=D\left(\theta_{\mathrm{i}}-\theta_{\mathrm{retro}}\right)\cdot\cos\left|\theta_{\mathrm{i}}+\theta_{\mathrm{r}}\right|$$

Based on Eq., the calculated far-field patterns (i.e., the real receiving signals) for corresponding retroreflective angles are represented by the blue lines in **Fig. S6b-d**. A comparison with the simulation results (red lines) shows that the theoretical and simulated receiving signals are in good agreement, validating the accuracy of the proposed method.

S8: The experimental results of abnormal spatial-harmonic reflection

The experimental results (*S*_21_ and *S*_11_) of abnormal harmonics reflection of the designed metasurface are shown as **Fig. S7**.

S9: The excitation of SSPs

The Spoof Surface Plasmon Polaritons (SSPs) are a kind of surface wave (SW) that propagates in the horizontal direction on the interface, while in the vertical direction appears as an exponentially decaying evanescent wave. According to the match of the dispersion, the transformation from propagating waves in radiation space to SWs in the interface can be achieved, as shown in **Fig. S8**. Utilizing the same four meta-atoms in a period as the three-channel retroreflector in this paper, the SSPs exciter can be achieved adding a propagation area. When the incident wave is illuminated on the excitation area vertically, the incident wave will be transformed as SSPs, and propagating along the propagation area. The simulation is performed in COMSOL with periodic boundary in the *y* direction.

Within the SH-GSL framework, surface plasmons arise naturally when an effective in-plane wavevector corresponding to the spatial harmonic exceeds *k*_0_, making its nominal reflection (or refraction) angle imaginary with real part 90° and producing an evanescent field along the *z* direction. In other words, the metasurface supplies the necessary lateral momentum |Δ*k_∥_* + 2*π*l/*Np*| > *k*_0_ to match the surface-plasmon dispersion. The key difference in our SH-GSL approach is that we can choose the fundamental compensation wave vector Δ*k_∥_* to be below *k*_0_ (so the 0th-order harmonic does not excite surface waves), while higher-order spatial harmonics (l ≠ 0) satisfy |Δ*k_∥_* + 2*π*l/*Np*| > *k*_0_ and thus selectively excite plasmons. This extends surface-plasmon coupling to higher-order channels without requiring an excessively large fundamental gradient.

For example, we design two metasurfaces under normal incidence:

(1). Δ*k_∥_* = 0.84*k*_0_ with supercell period *P* = (3 + 3)*p* (**Fig. S9a**). Here, the 0th-order harmonic cannot excite a surface wave, but the +1st and −3rd spatial harmonics lie in the plasmonic region and are excited (**Fig. S9b**).

(2). Δ*k_∥_* = 0.63*k*_0_ with supercell period *P* = (4 + 4)*p* (**Fig. S9c**). Similarly, the fundamental harmonic is sub-threshold while the +1st and −3rd spatial harmonics exceed *k*_0_ and excite surface plasmons (**Fig. S9d**).

These examples demonstrate that SH-GSL enables multi-channel near-field/far-field functionality: one can excite higher-order surface modes while avoiding the challenges of implementing very large phase gradients for the fundamental. This extension of surface-plasmon excitation beyond the fundamental gradient scenario in Sun et al. was not described previously.

S10: The generalized refraction validation

We have designed and simulated a transmissive metasurface operating at 10 GHz. The meta-atom consists of a three-layer dielectric (dielectric constant *ε*_r_ = 2.65, layer thickness *d* = 1.5 mm) with metal structures (strip width *w* = 0.2 mm, varying radius *r* = 1.15-4.6 mm) to achieve full 2*π* phase coverage in transmission (**Fig. S13**). The period of the meta-atom is *p* = 10 mm.

1. Transmissive Beam Splitters

- Dual-beam splitter (**Fig. S14a**)

Using SH-GSL, we select Δ*k_∥_* = 0.75*k*_0_ with supercell *P* = (2+2)*p*. The farfield simulation (**Fig. S14b**) and the field distribution on the *xoz* plane (**Fig. S14c**) via COMSOL show two transmitted beams at the predicted angles, matching the theoretical splitting angles from SH-GSL.

- Quad-beam splitter (**Fig. S14d)**

With Δ*k_∥_* = 0.375*k*_0_ and *P* = (4 + 4)*p*, simulations (**Fig. S14e** and **f**) again exhibit four transmitted beams at the angles calculated by SH-GSL, confirming the design principle.

1. Transmissive Negative Refraction

- Two-channel negative refraction (**Fig. S15a**)

We design Δ*k_∥_* = 1.50*k*_0_ with *P* = (1 + 1)*p*. Under normal or specified oblique incidence, the transmitted beam is refracted negatively at the angle predicted by SH-GSL (**Fig. S15a-c**), with a clean far-field lobe and high efficiency, analogous to the perfect three-channel retroreflection in reflection.

- Four-channel negative refraction (**Fig. S15d**)

With Δ*k_∥_* = 0.75*k*_0_ and *P* = (2 + 2)*p* (same supercell arrangement as the four-beam splitter), simulations (**Fig. S15d-i**) show that for the two central incidence angles (±22°), negative refraction is achieved with reasonable efficiency and purity; for other channels, additional unwanted harmonics appear, reducing purity. This behavior matches SH-GSL predictions and parallels the imperfect five-channel retroreflector performance.

These full-wave simulation results validate that the SH-GSL holds in transmission: by choosing appropriate Δ*k_∥_* via the metasurface phase profile and supercell period, one can selectively activate desired transmitted harmonics for beam splitting or negative refraction.

S11: Experimental validation of dual-beam splitter via single horn setup

We have performed single-horn *S*_11_ measurements on the dual-beam splitter to directly assess suppression of the mirror mode at normal incidence. Specifically, with the horn fixed normal to the metasurface, we recorded *S*_11_ over an angular range from –20° to +20°. The measured *S*_11_ values were normalized using the maximum value from the farfield beam-splitting data (Fig. 4l). The resulting curve (**Fig. S16**) shows that around 0° incidence (the range of the mirror reflection), *S*_11_ is essentially zero, indicating that the mirror reflection is effectively suppressed by the metasurface under normal incidence.

Supplementary Figures


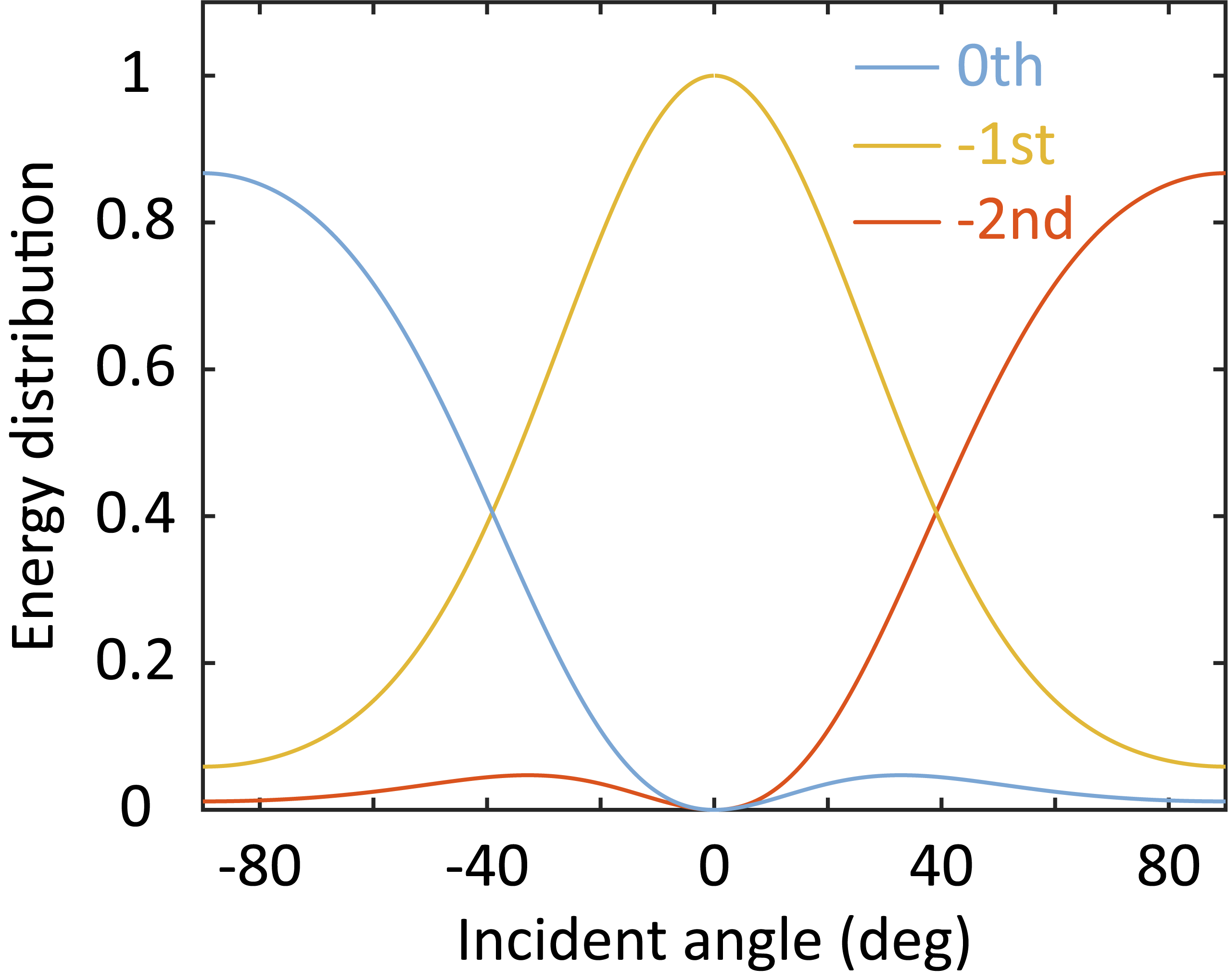


**Fig. S1: The energy distribution of spatial harmonics for the designed three-channel retroreflection.** The energy distribution of the mirror mode (−1st, yellow line) is at its maximum at 0° incidence, and it gradually decreases as the angle of incidence increases. The energy distribution of the 0th (blue line) and −2nd (red line) spatial harmonics is respectively distributed on both sides, corresponding to the retroreflective channel.





**Fig. S2: The analysis of spatial harmonics energy distribution.** **a** The gradient metasurface for three-channel retroreflection. **b** The repeating-cell metasurface for abnormal single-side reflection. **c** The gradient metasurface for five-channel retroreflection. **d** The repeating-cell metasurface for five-channel retroreflection. **e** The gradient metasurface for dual-beam splitting. **f** The repeating-cell metasurface for dual-beam splitting. **g** The gradient metasurface for quad-beam splitting. **h** The repeating-cell metasurface for quad-beam splitting.


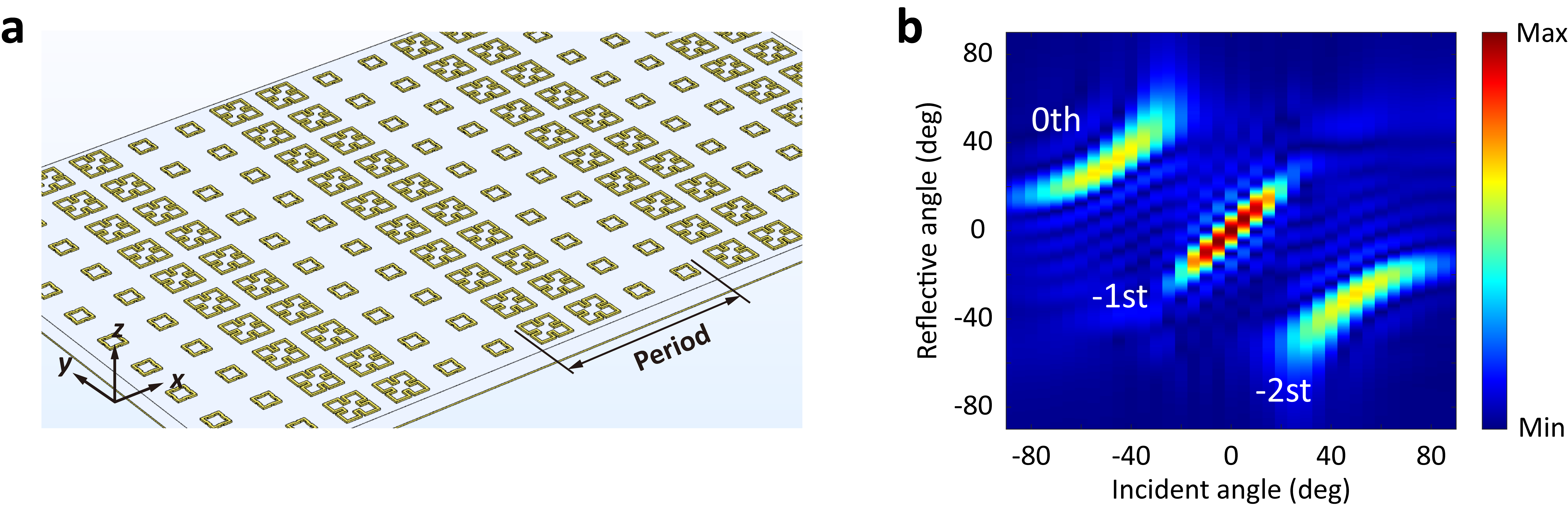


**Fig. S3: The structure of the metasurface and the simulation results. a**The structure of the designed metasurface. Each supercell integrates two ‘0’ and ‘*π*’ meta-atoms with period *P* = 4*p*. **b** The simulation results in abnormal harmonics reflection.


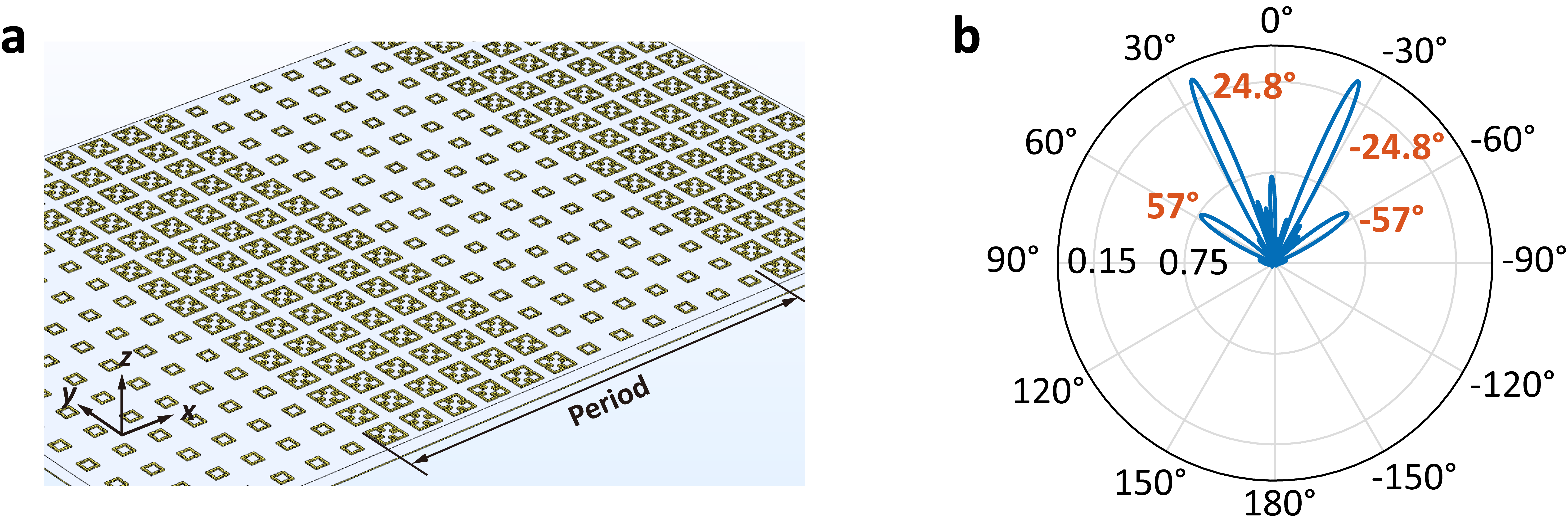


**Fig. S4: The structure and simulation results of the quad-beam splitter.** **a** The structure of the quad-beam splitter. Each supercell integrates six ‘0’ and six ‘*π*’ meta-atoms with period *P* = 12*p*. **b** The far-field pattern calculated by simulation.


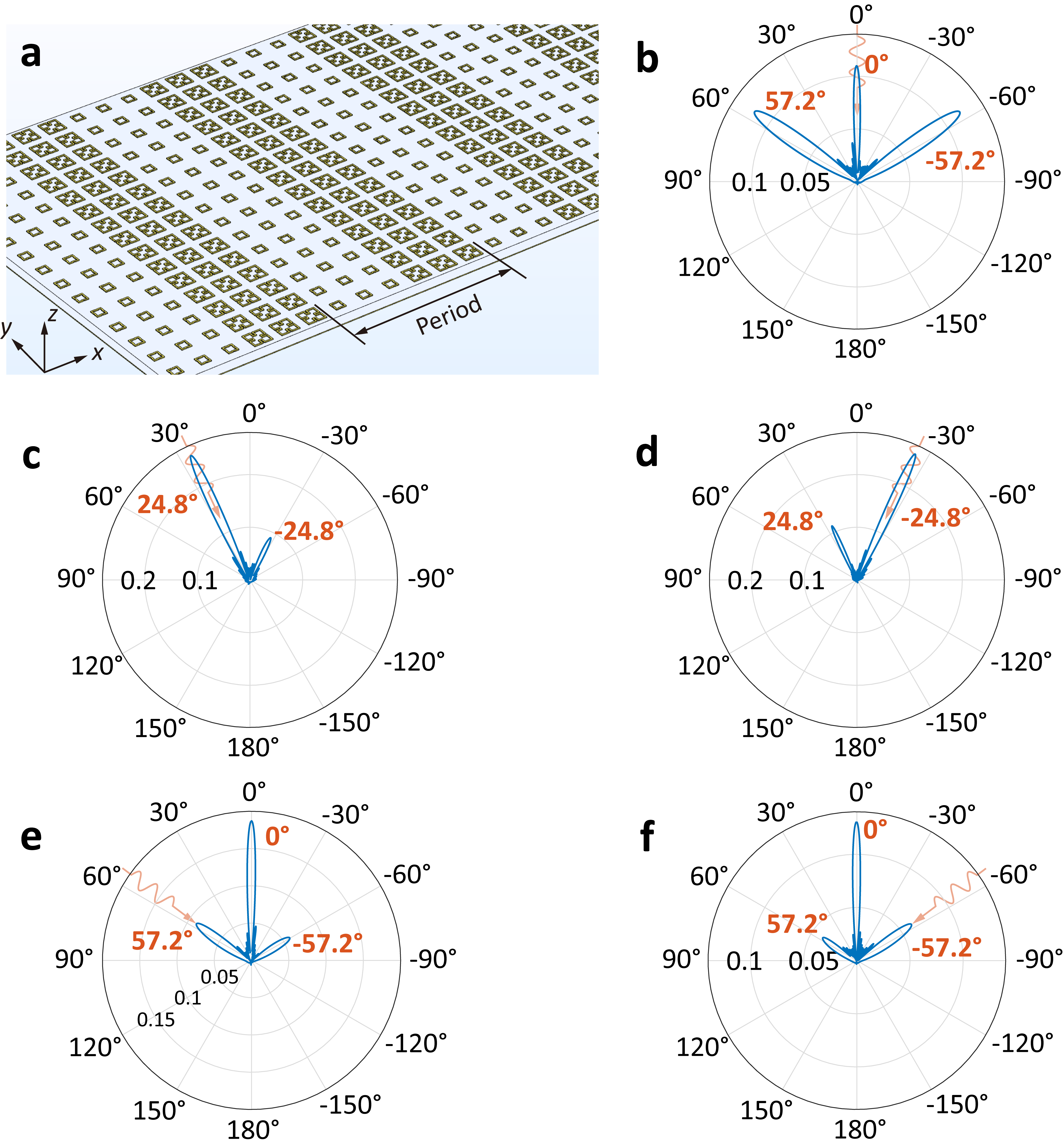


**Fig. S5: The designed five-channel retroreflector.** **a** The structure of the five-channel retroreflector. Each supercell integrates three ‘0’ and three ‘*π*’ meta-atoms with period *P* = 6*p*. **b**, **c**, **d**, **e**, and **f**  represent the case of incidence at 0° (l = −1), 24.8° (l = −2), −24.8° (l = 0), 57.2° (l = −3), and −57.2° (l = +1).


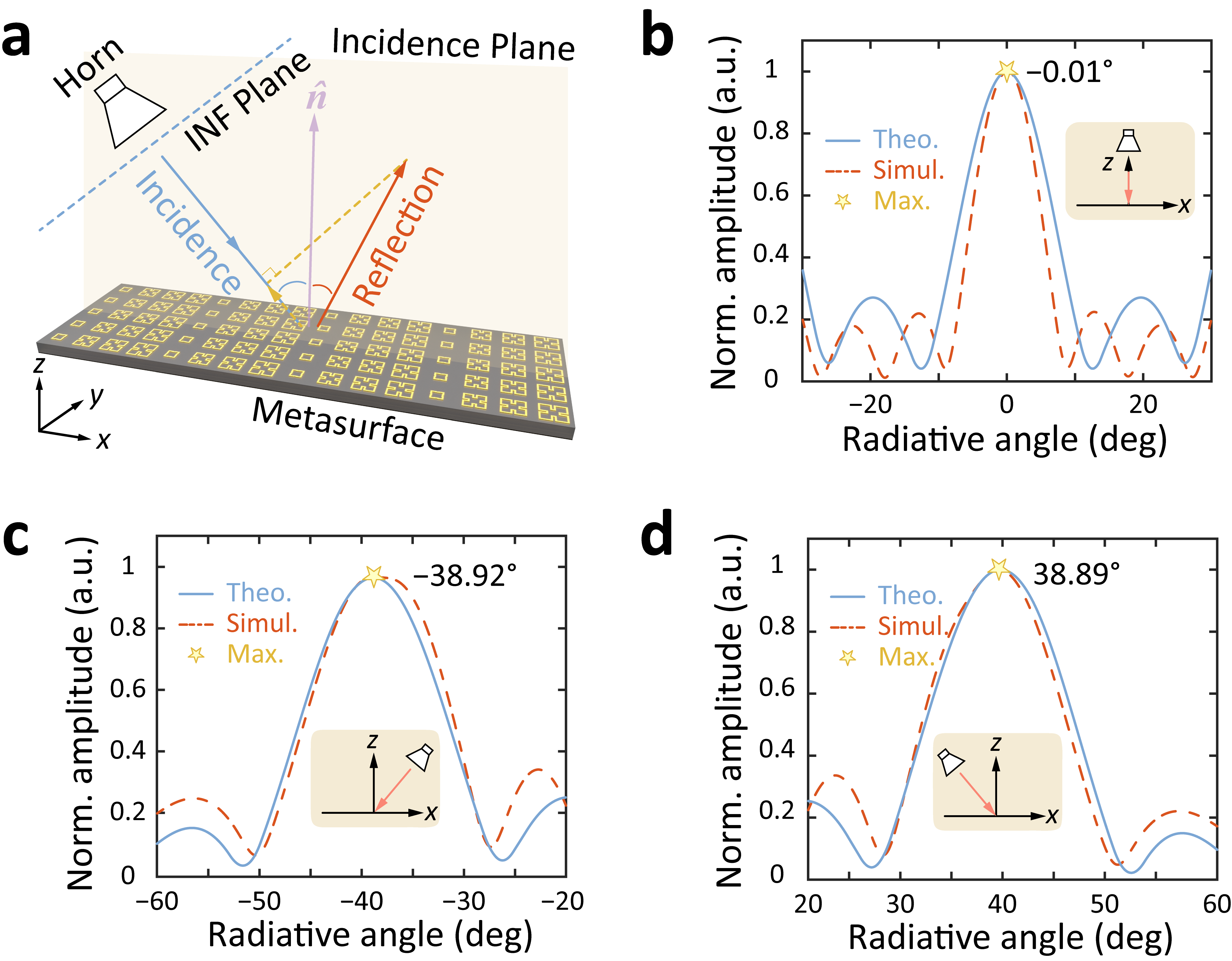


**Fig. S6: The schematic of the method for calculating the far-field radiation.** **a** The illustration of the method. The far-field radiation results of theory and simulation under **b** 0°, **c** −39°, and **d** 39° incidence. The different lines represent the results of theory (blue solid lines) and simulation (red dotted lines). The inserts of **b**, **c**, and **d** represent the incidence of different retroreflections, and the stars of **b**, **c**, and **d** represent the position of max reflection, i.e., retroreflective angles.


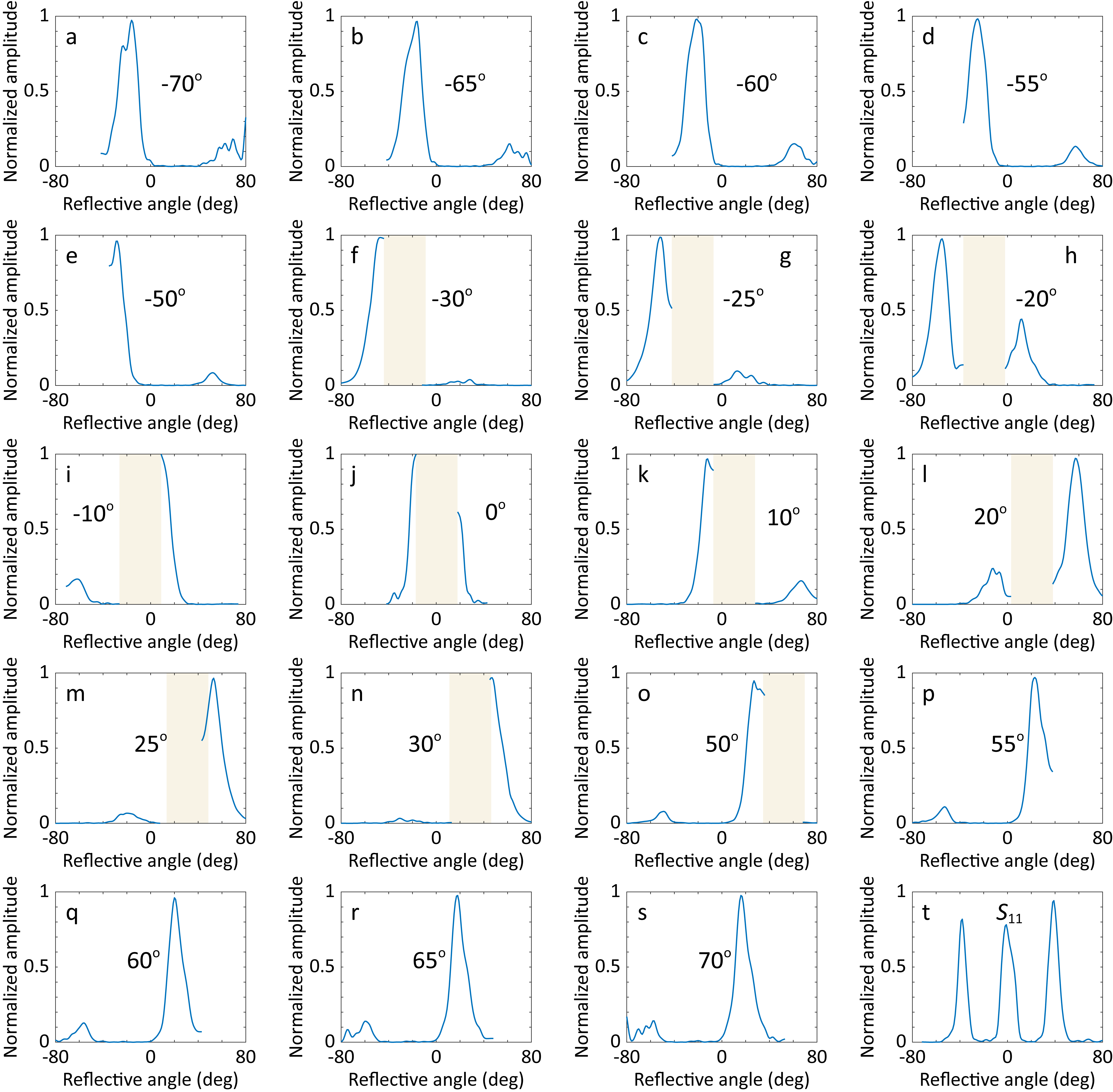


**Fig. S7: The experimental results for abnormal spatial-harmonic reflection.** **a**-**s**  The measured *S*_21_ of the designed metasurface. **t**The measured *S*_11_ of the designed metasurface.


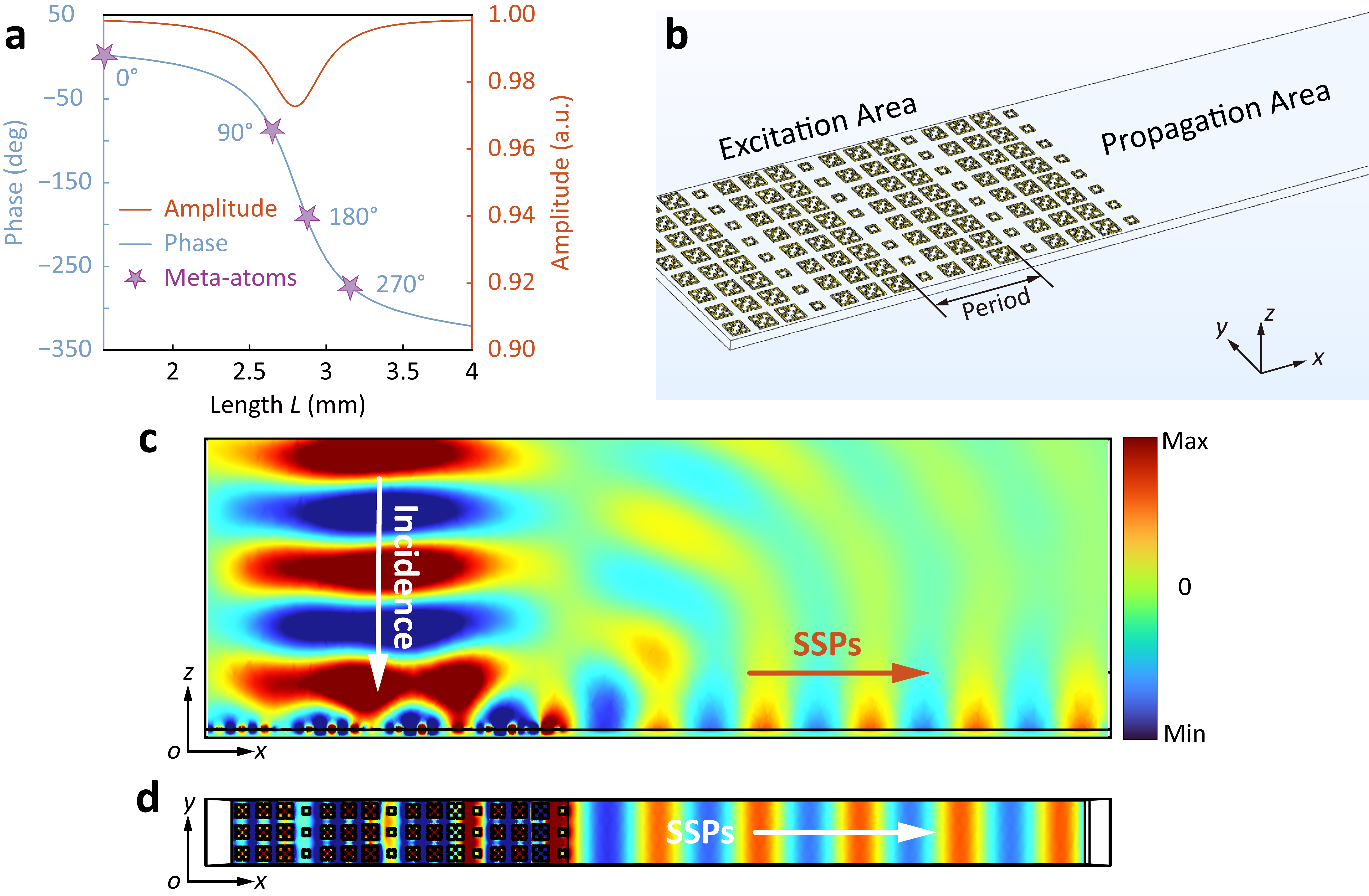


**Fig. S8: The schematic of the SSP excitator. a** The distribution of the meta-atoms, and the phase gradient is *π*/2, which is the same as the retroreflector in this paper. **b** The illustration of the SSP exciter, which is composed of the excitation area and the propagation area. **c** The distribution of the electric field in the *xoz* plane, with high efficiency. **d** The distribution of the electric field in the *xoy* plane.


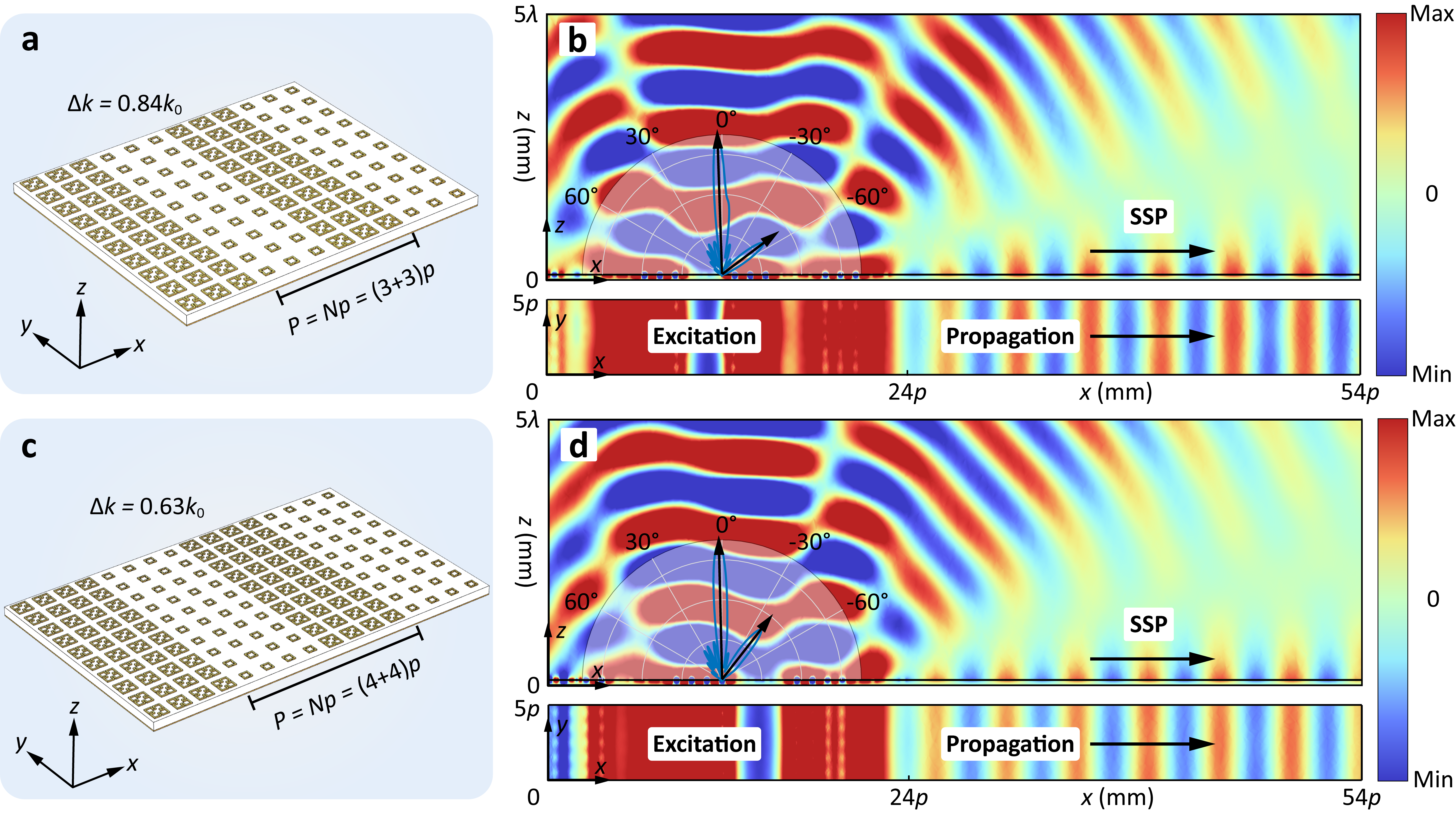


**Fig. S9: Excitation of surface waves by high-order spatial harmonics via SH-GSL. a** The designed metasurface with the compensation wave vector Δ*k_∥_* = 0.84*k*_0_, utilizing the repeating-cell metasurface with 3 + 3 meta-atoms. **b** The simulated field distribution of *xoz* and *xoy* via COMSOL. **c** The designed metasurface with the compensation wave vector Δ*k_∥_* = 0.63*k*_0_, utilizing the repeating-cell metasurface with 4 + 4 meta-atoms. **d** The simulated field distribution of *xoz* and *xoy* via COMSOL.


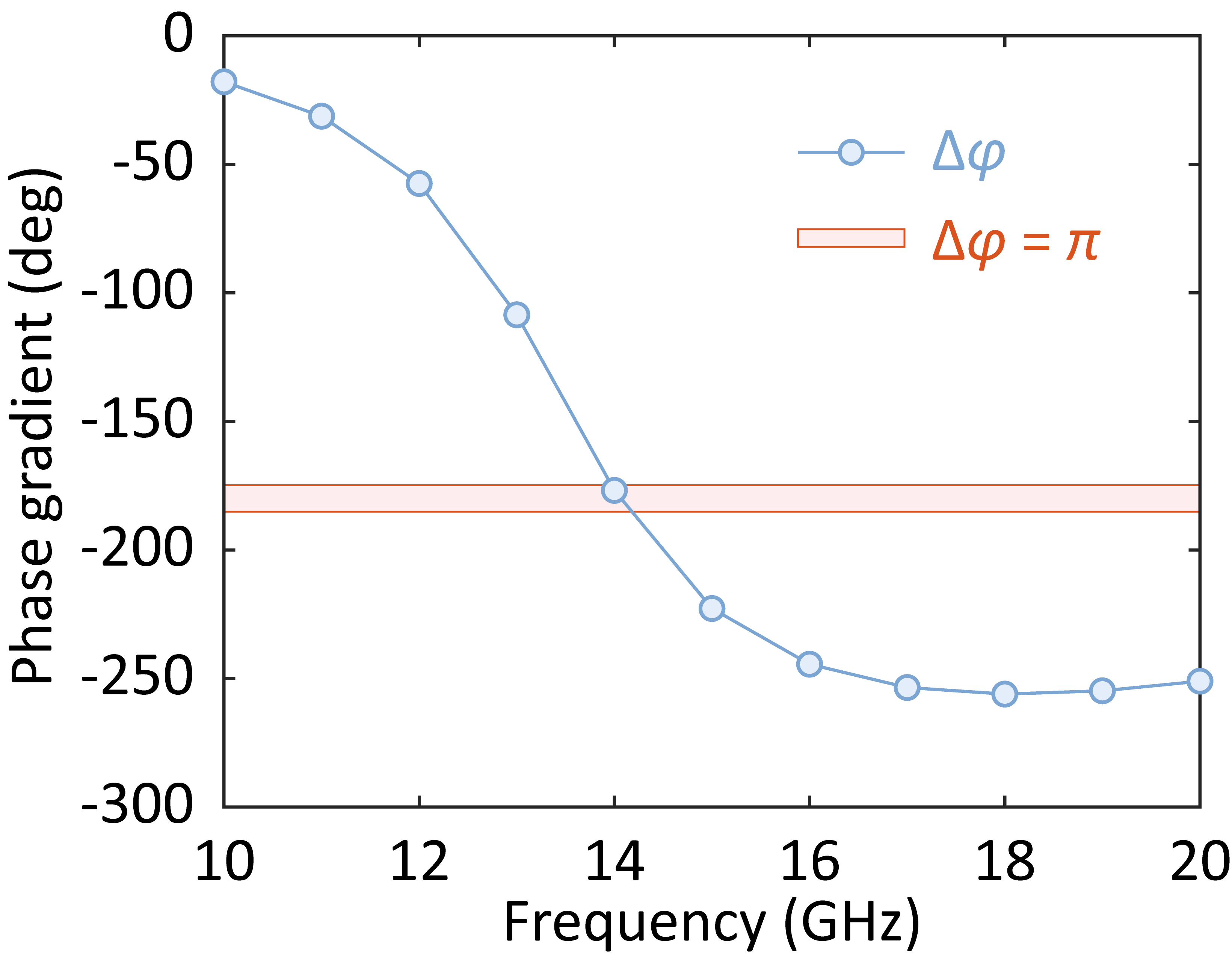


**Fig. S10: The phase difference of repeating-cell metasurface at different frequency.** The designed operating frequency is 14 GHz with the phase difference of *π*.


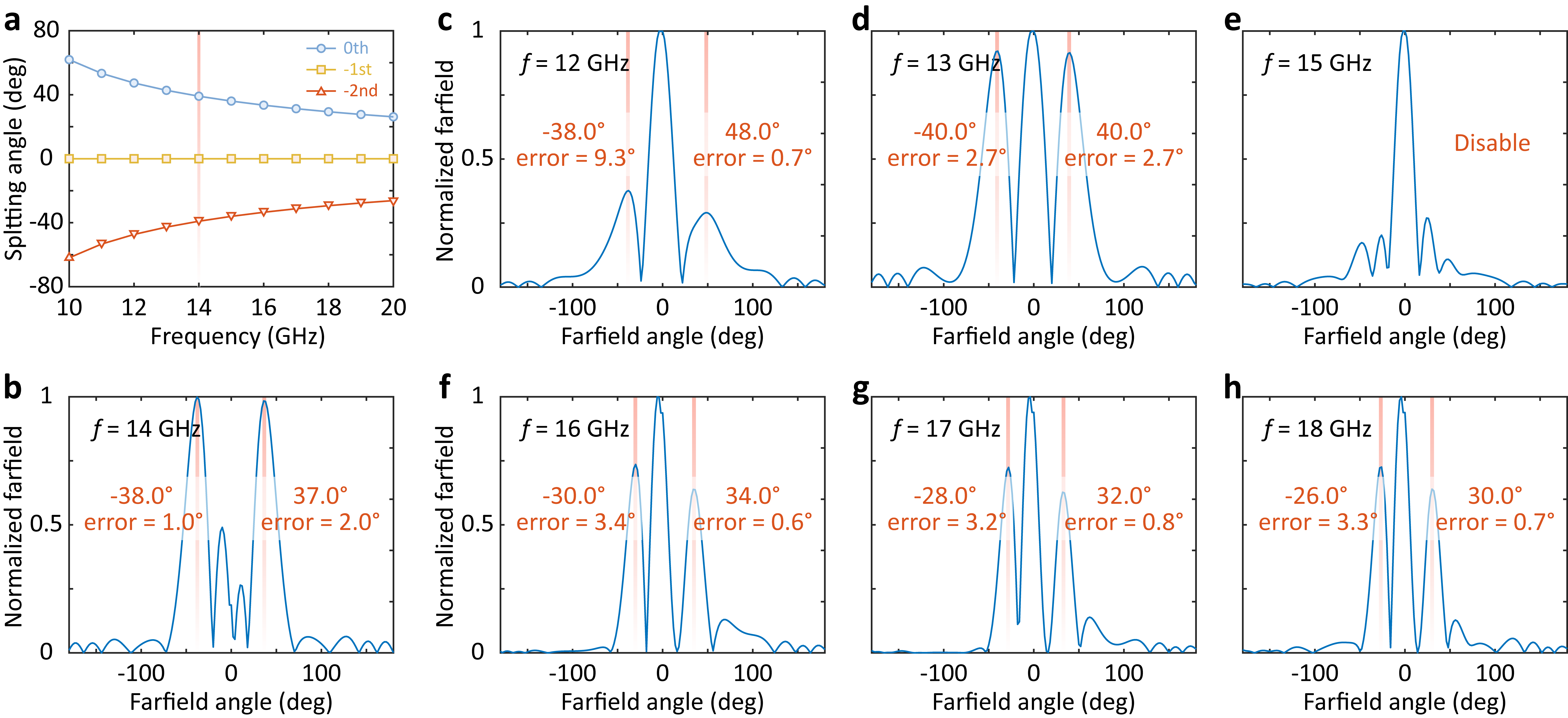


**Fig. S11:** **Broadband analysis of the designed dual-beam splitting.** **a** The theoretical values of the beam splitting angle within the broadband range. **b** The simulated farfield at 14 GHz with the theoretical values of ±39°. **c** The simulated farfield at 12 GHz with the theoretical values of ±47.3°. **d** The simulated farfield at 13 GHz with the theoretical values of ±42.7°. **e** The simulated farfield at 15 GHz with the theoretical values of ±36°. **f** The simulated farfield at 16 GHz with the theoretical values of ±33.4°. **g** The simulated farfield at 17 GHz with the theoretical values of ±31.2°. **h** The simulated farfield at 18 GHz with the theoretical values of ±29.3°.


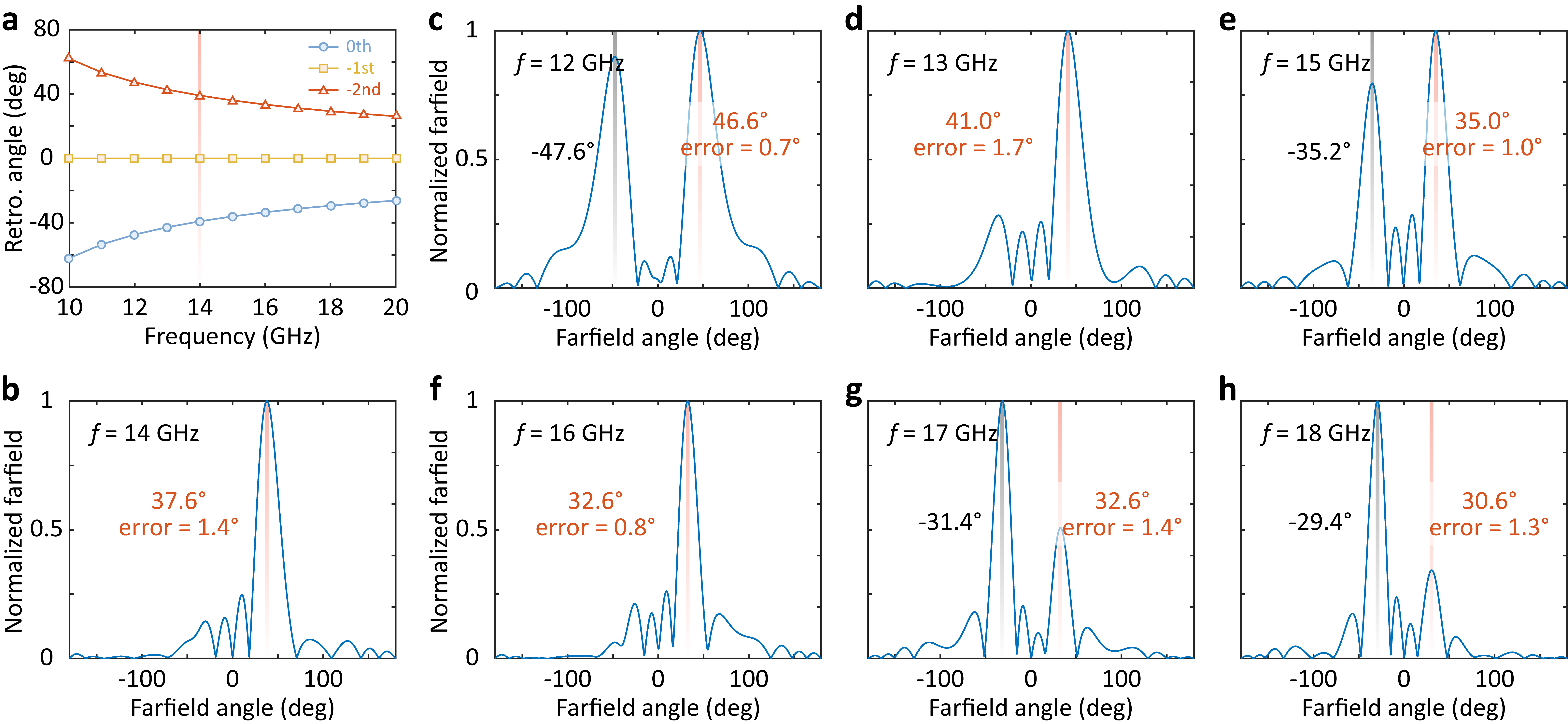


**Fig. S12:** **Broadband analysis of the designed three-channel retroreflector.** **a** The theoretical values of the retroreflective angle within the broadband range. **b** The simulated farfield at 14 GHz with the theoretical values of ±39°. **c** The simulated farfield at 12 GHz with the theoretical values of ±47.3°. **d** The simulated farfield at 13 GHz with the theoretical values of ±42.7°. **e** The simulated farfield at 15 GHz with the theoretical values of ±36°. **f** The simulated farfield at 16 GHz with the theoretical values of ±33.4°. **g** The simulated farfield at 17 GHz with the theoretical values of ±31.2°. **h** The simulated farfield at 18 GHz with the theoretical values of ±29.3°.


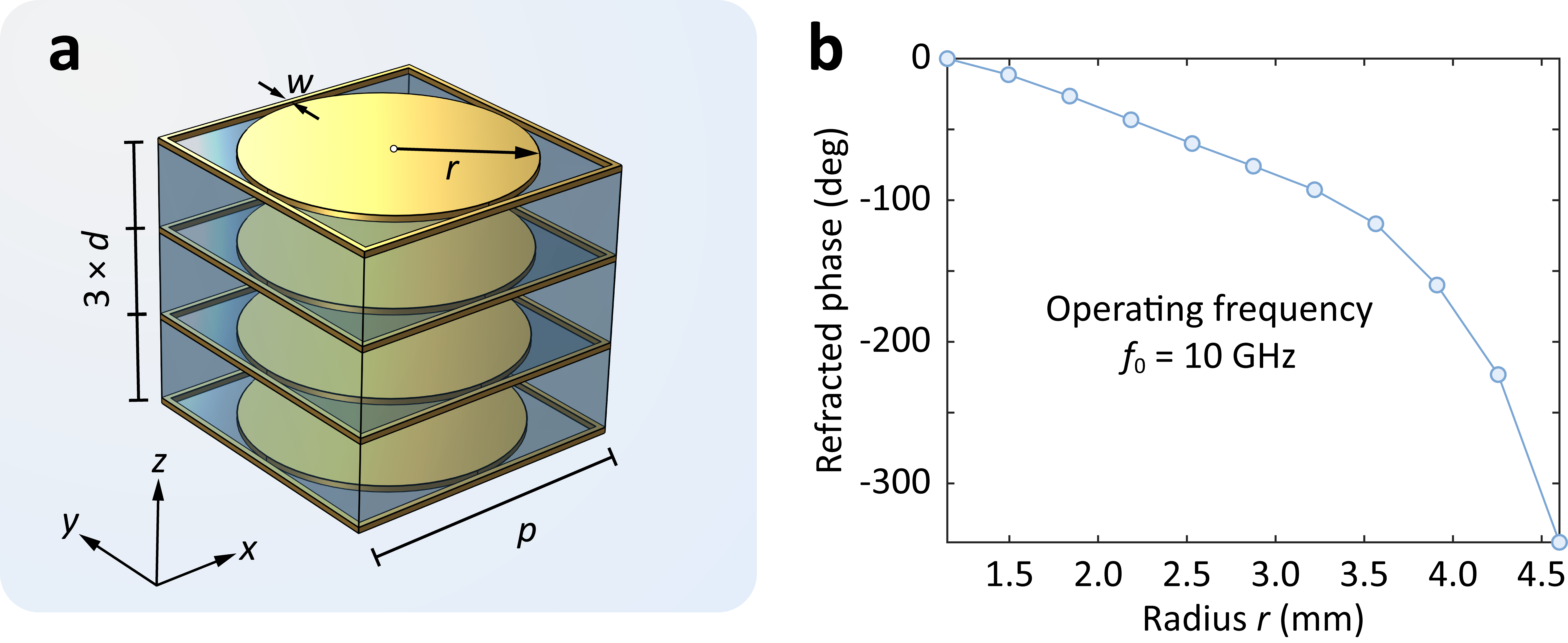


**Fig. S13: The design of the transmissive metasurface at 10 GHz. a** The schematic of the meta-atom. **b** The transmissive phase of the meta-atom.


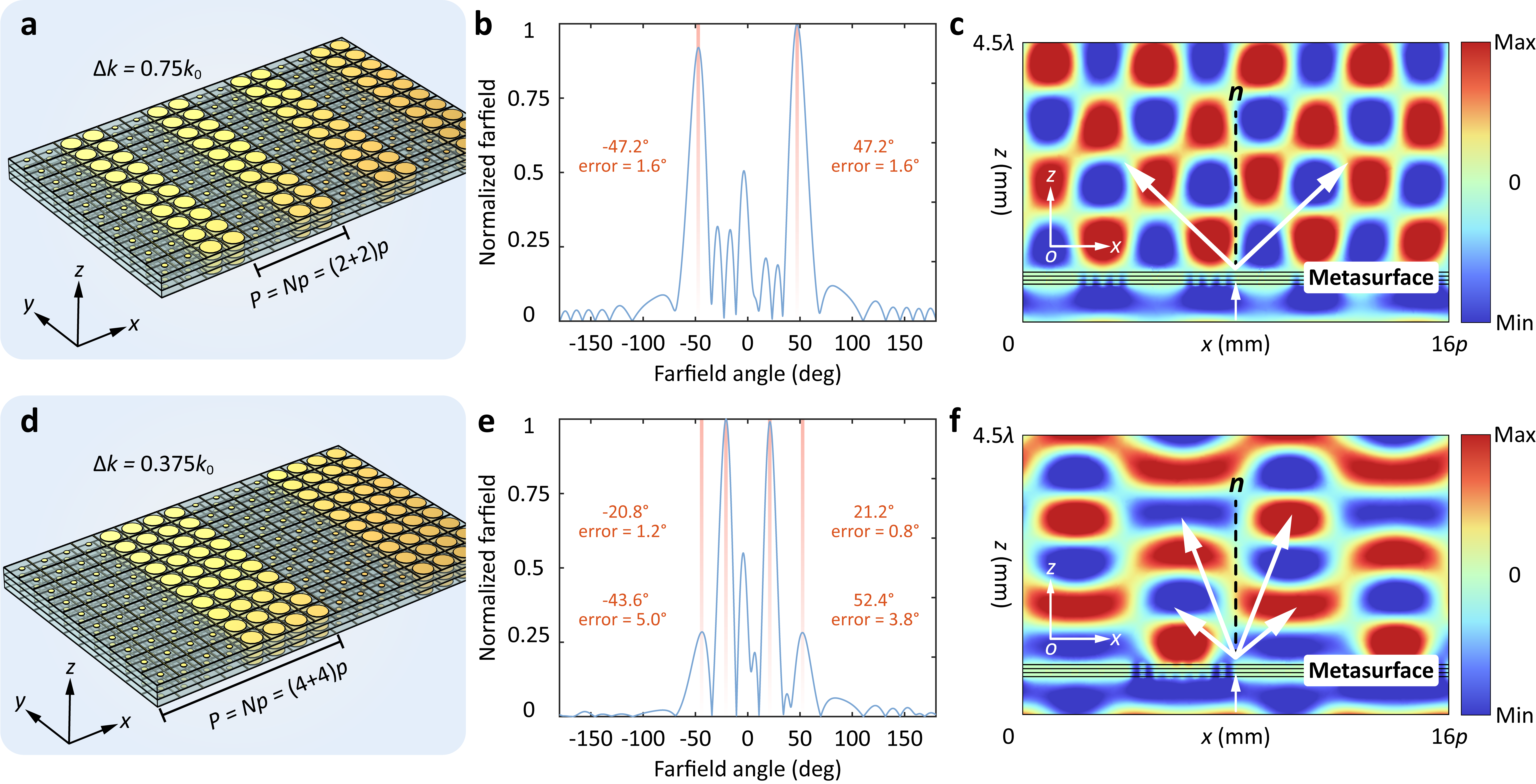


**Fig. S14: The beam splitting via the transmissive metasurface. a** The designed dual-beam splitter with the compensation wave vector Δ*k_∥_* = 0.75*k*_0_, utilizing the repeating-cell metasurface with 2 + 2 meta-atoms. **b** The farfield pattern of the dual-beam splitter with the theoretical values of ±48.6°. **c** The field distribution on the *xoz* plane. **d** The designed quad-beam splitter with the compensation wave vector Δ*k_∥_* = 0.375*k*_0_, utilizing the repeating-cell metasurface with 4 + 4 meta-atoms. **e** The farfield pattern of the quad-beam splitter with the theoretical values of ±22.0° and ±48.6°. **f** The field distribution on the *xoz* plane.


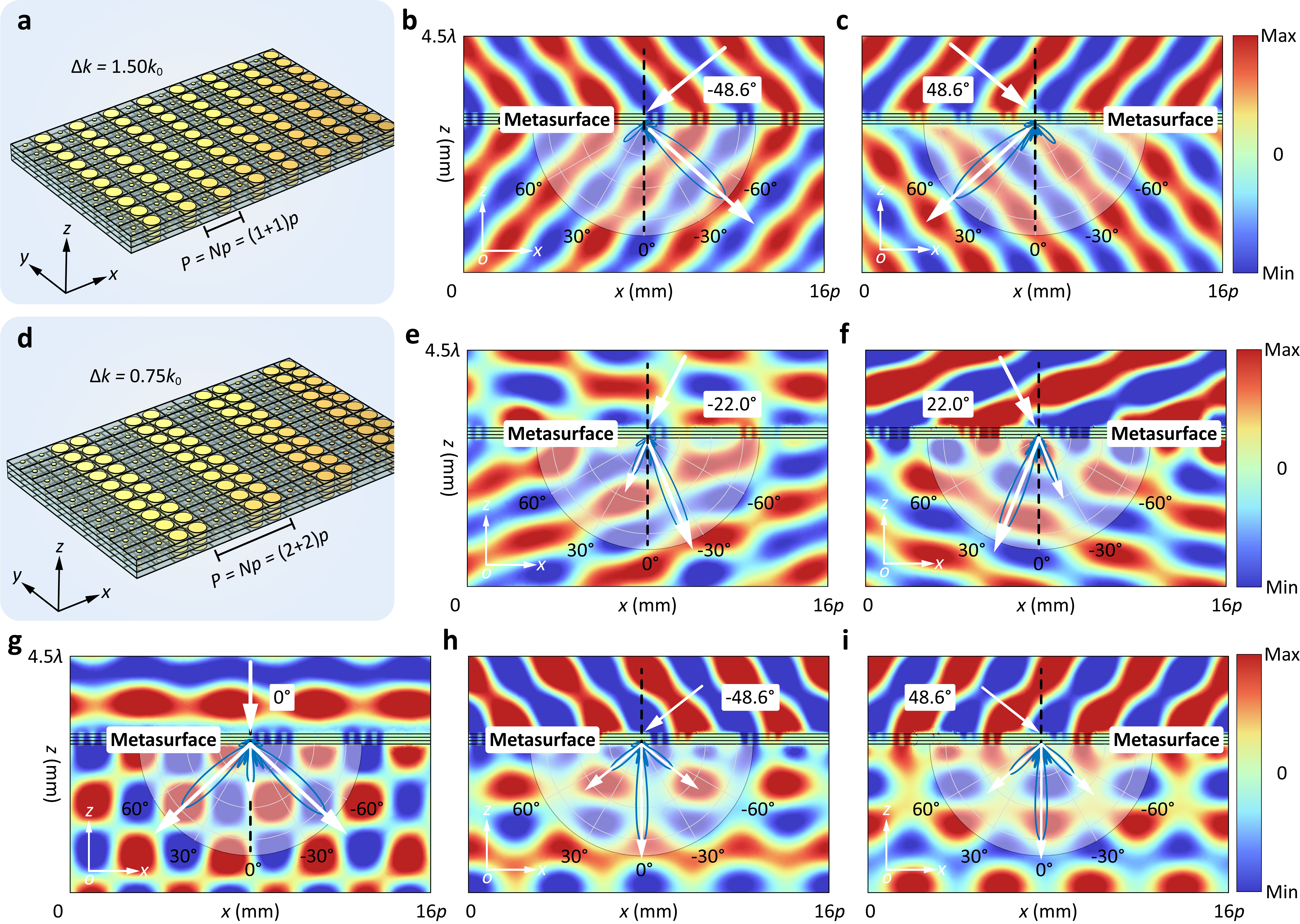


**Fig. S15: The negative refraction via the transmissive metasurface. a** The designed two-channel negative refraction with the compensation wave vector Δ*k_∥_* =1.50*k*_0_, utilizing the repeating-cell metasurface with 1 + 1 meta-atoms. **b-c** The field distribution on the *xoz* plane and the farfield pattern of the two-channel negative refraction at −48.6° and +48.6°.**d** The designed four-channel negative refraction with the compensation wave vector Δ*k_∥_* = 0.75*k*_0_, utilizing the repeating-cell metasurface with 2 + 2 meta-atoms. **e-f** The field distribution on the *xoz* plane and the farfield pattern of the four-channel negative refraction at −22.0°, +22.0°, 0°,−48.6° and +48.6°.


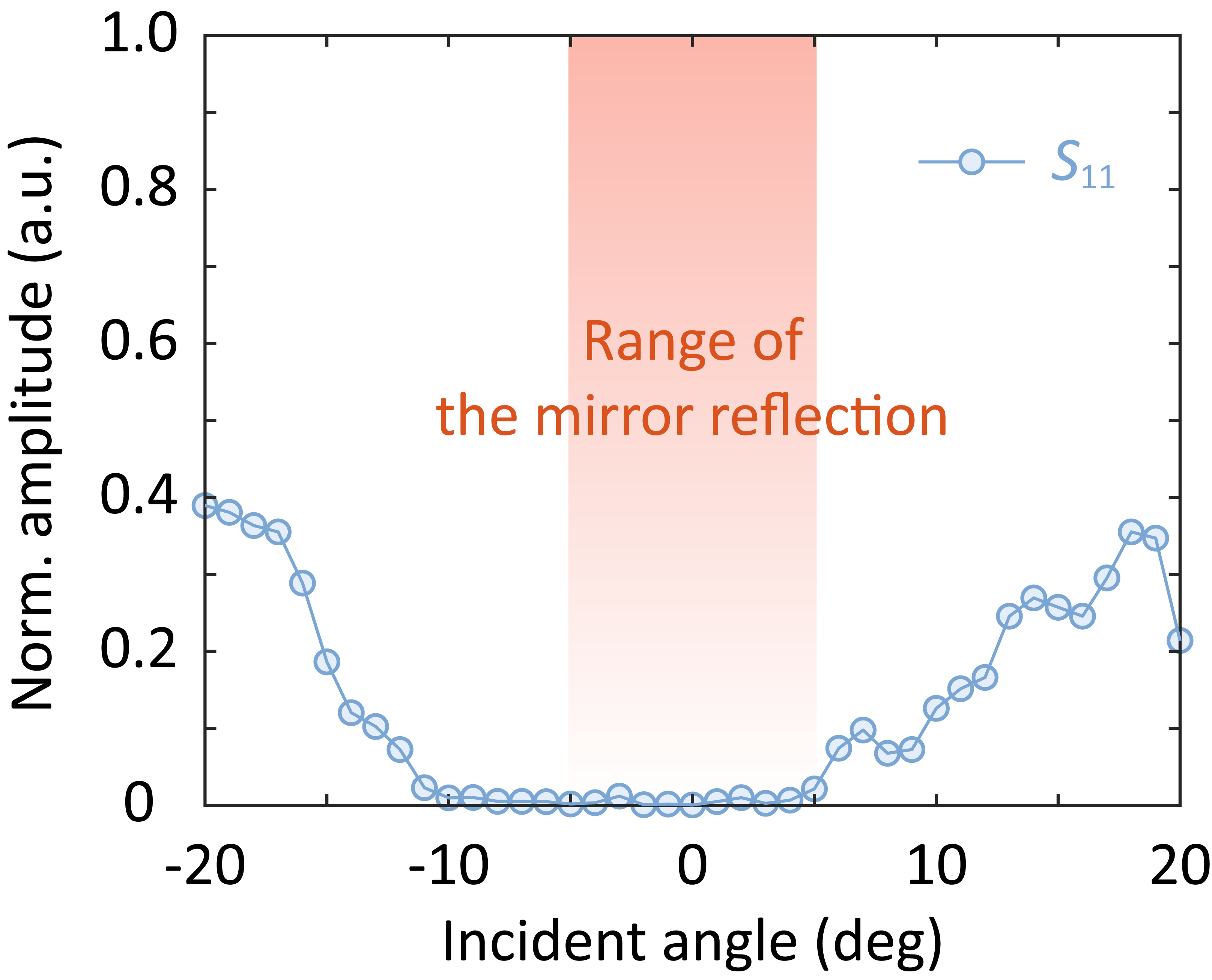


**Fig. S16: The measurement result of the dual-beam splitter via the single-horn setup.** Within the range of the mirror reflection, *S*_11_ is essentially zero.
